# Time-Resolved Excited-State Analysis of Molecular Electron Dynamics by TDDFT and Bethe–Salpeter Equation Formalisms

**DOI:** 10.1021/acs.jctc.1c00211

**Published:** 2021-09-06

**Authors:** P. Grobas Illobre, M. Marsili, S. Corni, M. Stener, D. Toffoli, E. Coccia

**Affiliations:** † Dipartimento di Scienze Chimiche e Farmaceutiche, Universitá di Trieste, via L. Giorgieri 1, Trieste 34127, Italy; ‡ Dipartimento di Scienze Chimiche, Universitá di Padova, via Marzolo 1, Padova 35131, Italy; § CNR Istituto di Nanoscienze, via Campi 213/A, Modena 41125, Italy

## Abstract

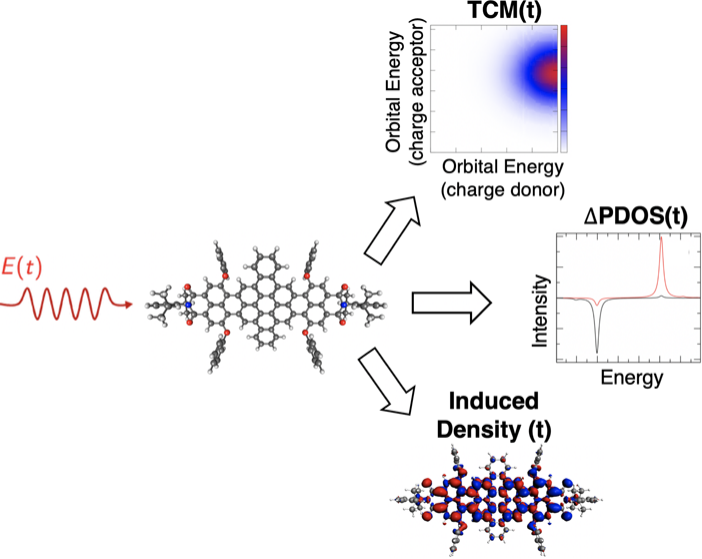

In this work, a theoretical and computational set of tools to study and analyze time-resolved electron dynamics in molecules, under the influence of one or more external pulses, is presented. By coupling electronic-structure methods with the resolution of the time-dependent Schrödinger equation, we developed and implemented the time-resolved induced density of the electronic wavepacket, the time-resolved formulation of the differential projection density of states (ΔPDOS), and of transition contribution map (TCM) to look at the single-electron orbital occupation and localization change in time. Moreover, to further quantify the possible charge transfer, we also defined the energy-integrated ΔPDOS and the fragment-projected TCM. We have used time-dependent density-functional theory (TDDFT), as implemented in ADF software, and the Bethe–Salpeter equation, as provided by MolGW package, for the description of the electronic excited states. This suite of postprocessing tools also provides the time evolution of the electronic states of the system of interest. To illustrate the usefulness of these postprocessing tools, excited-state populations have been computed for HBDI (the chromophore of GFP) and DNQDI molecules interacting with a sequence of two pulses. Time-resolved descriptors have been applied to study the time-resolved electron dynamics of HBDI, DNQDI, LiCN (being a model system for dipole switching upon highest occupied molecular orbital–lowest unoccupied molecular orbital (HOMO–LUMO) electronic excitation), and Ag_22_. The computational analysis tools presented in this article can be employed to help the interpretation of fast and ultrafast spectroscopies on molecular, supramolecular, and composite systems.

## I Introduction

Recent impressive developments in both tunable ultrafast sources and in efficient algorithms for the propagation of the time-dependent Schrödinger equation, opened the way to the control of chemical reactions and electron processes triggered by ultrashort light pulses.^1,2^ On the one hand, molecular vibrations, which are the underlying elementary dynamical steps of any chemical reaction, are best controlled using femtosecond (1 fs = 10^–15^ s) light pulses.^1^ On the other hand, thanks to the major development in the technology of coherent light sources in the last 20 years, one can nowadays generate coherent light pulses in the attosecond (1 as = 10^–18^ s) time scale.^3−5^ Attosecond pulses in the UV spectral region were first used to investigate ultrafast electron dynamics in atoms and small molecules,^6−8^ and are nowadays also employed to investigate electron dynamics in molecules of biological relevance^2,9^ and at the nanoscale,^10^ where electron transfer may play a critical role.

Theoretical modeling of fast and ultrafast phenomena occurring in systems stimulated by femto- and attosecond light is needed to rationalize, interpret, and predict experimental outcomes.^11−18^ Extending electronic-structure methods to the time domain is an essential step to reduce the gap with the experimental realm. Time-dependent wavefunction^16,19−31^ and DFT/ time-dependent density-functional theory (TDDFT)^32−34^ methods have been successfully formulated over the years. In particular, descriptors to study charge evolution in metal clusters and nanosystems under the influence of an explicit electromagnetic field have been implemented within the real-time TDDFT framework.^35−39^ Such descriptors include the transition density,^40^ the projected density-of-states of molecular orbitals (PDOS),^41^ and the transition contribution map (TCM),^42^ which are adopted to describe the possible plasmonic behavior of the electron dynamics.

In this work, we propose a general approach that couples the time propagation of an electronic wavepacket under the influence of one (or multiple) external field(s), which is implemented in the in-house WaveT code,^43−45^ with a TDDFT (by means of ADF package^41^) and the GW/Bethe–Salpeter equation (BSE) (by means of MolGW code^46^) description of the excited states. In recent years, GW/BSE has been extensively applied to molecular excited-state properties.^47−59^ We have implemented various postprocessing tools with the aim to characterize and describe the electron dynamics under different conditions, in particular here we present how to compute, in a time-resolved manner and in a molecular framework the following: (1) the populations of electronically excited states, also by exploiting transition dipole moments between excited states; (2) the induced density; (3) the differential PDOS of molecular orbitals (ΔPDOS); and (4) the TCM, starting from the definition in ref (42). These tools are of general purpose since they can be applied to gain insights into a wide range of physical processes and systems, as multiple-pulse time-resolved spectroscopies on single molecules,^60^ pump-probe experiments,^1,61−67^ molecular nanoplasmonics,^15,68−75^ plasmon-assisted catalysis,^76−78^ etc. Extension of tools such as PDOS and TCM, originally defined in the frequency domain to the time domain is essential to simulate and interpret time-resolved ultrafast experiments in molecular and composite systems^2,72^ and to suggest novel routes of investigation.

**Figure 1:**
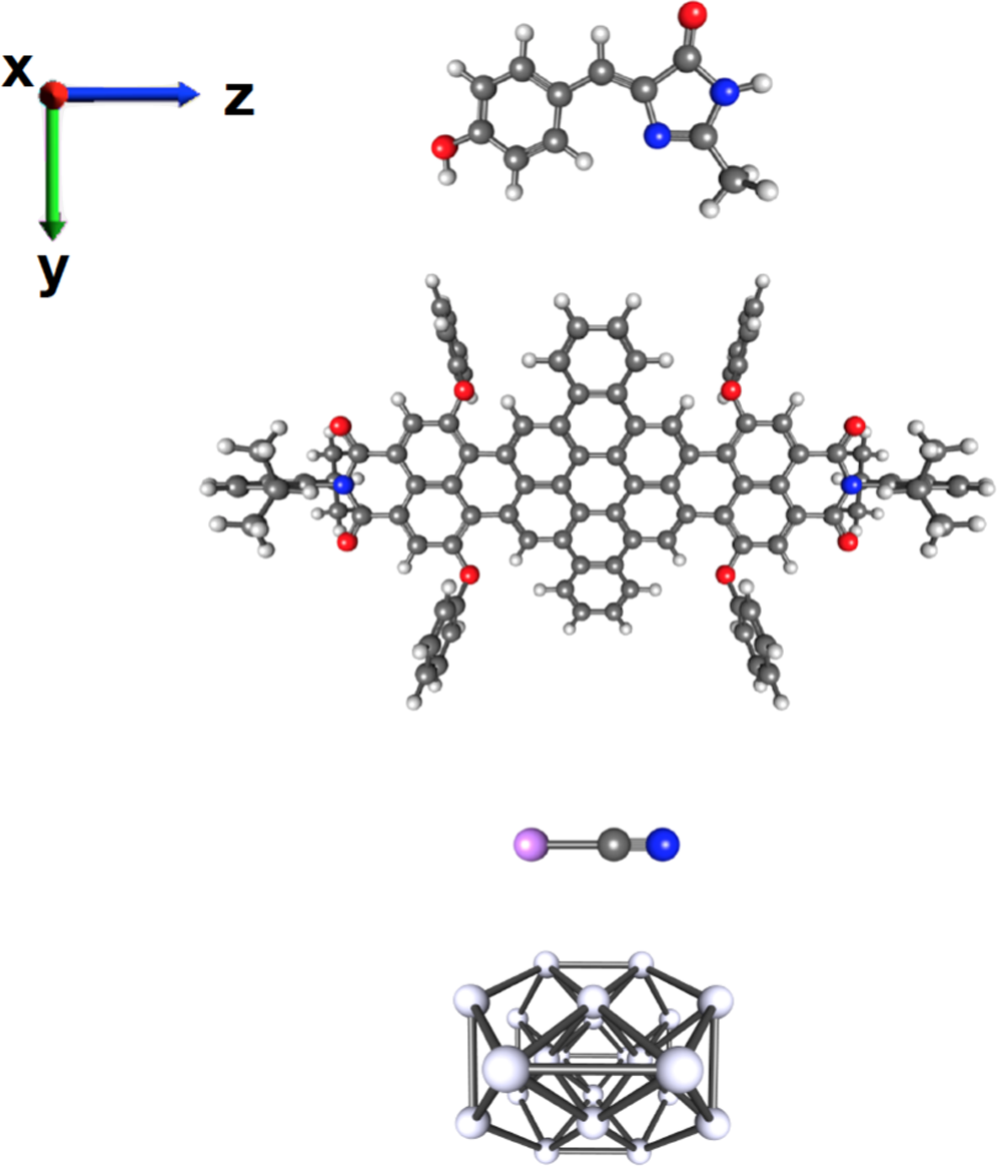
Ground-state structures of the systems investigated in the present work, from top to bottom: HBDI, DNQDI, LiCN, and Ag_22_. Color code: H, white; C, black; O, red; N, blue; Li, purple; and Ag, gray.

Here, we report the results of the application of TDDFT and GW/BSE postprocessing, i.e., populations, induced density, ΔPDOS, and TCM, on the HBDI molecule, which is the chromophore of the GFP protein,^79−91^ the DNQDI fluorophore, which has been used to study the interplay between electronic and vibrational quantum coherence,^92,93^ the LiCN molecule, which has been chosen as a computational model for dipole switching, as reported in the literature,^21,43,45,94,95^ and a small metal cluster Ag_22_, which is the prototype of systems with collective optical responses.^96^ The ground-state equilibrium structures of these systems are reported in Figure 1. In detail, visible pump–probe “experiments” have been simulated for HBDI and DNQDI, while the time evolution of the charge transfer in LiCN (triggered by a pulse resonant with the dipole-inverting excitation energy) has been characterized by means of the induced density, ΔPDOS, and TCM. Time-resolved TCM for Ag_22_ has been used to analyze the temporal evolution of the electron wavepacket under the influence of a pulse with a frequency corresponding to the maximum absorption. Our general goal is to provide a reliable and accurate computational protocol for the description of electron dynamics in molecules and nanosystems, based on TDDFT and GW/BSE, in view also of future applications in molecular nanoplasmonics.

The article is organized as follows: in Section II, we focus on the definition of the descriptors introduced above, computational details are collected in Section III, results are presented and discussed in Section IV, while in Section V, main outcomes are summarized and perspectives for future work are indicated.

## II Theory

In this section, starting from the definition of the electronically excited states in terms of TDDFT or GW/BSE eigenvectors, we describe the calculation of transition dipole moments between such excited-excited pairs, and the time-resolved formulation, at TDDFT and the GW/BSE level of theory, of induced density, ΔPDOS, and TCM.

### II.I Excited-State Formalism and the Calculation of Transition Dipole Moments

Both TDDFT and GW-BSE linear response problems can be recast in terms of an effective two-body Hamiltonian, which describes the correlated propagation of an excited electron–hole pair.^47^ In this framework, optical spectra are given in terms of the eigenvalues and eigenvectors of such two-body Hamiltonians and, in the limit of the approximation of their implementation (for example, the approximated *f*_*xc*_ kernel for TDDFT, and approximated d∑/d*G* for BSE, ∑, and *G* being the electronic self-energy and Green’s function, respectively^47^), the two theories directly provide excited-state energies and dipole matrix elements between the ground (|Φ_0_⟩) and excited electronic states. In this work, we further employ the TDDFT and BSE eigenvectors within a configuration-interaction singles ansatz^97^ for the excited states expressed as
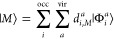
1where |Φ_*i*_^*a*^⟩ is the singly excited Slater determinant, with an electron moving from the occupied orbital *i* to the virtual one *a*, while *d*_*i*,*M*_^*a*^ are the linear coefficients of the expansion for the state |*M*⟩. In this work, the expansion coefficients, *d*_*i*,*M*_^*a*^, and the energies of the excited states, *E*_*M*_, are obtained by means of the ADF^41^ and MolGW^46^ packages.

Coefficients *d*_*i*,*M*_^*a*^ of eq 1 differ in full TDDFT and within the Tamm–Dankoff approximation (TDA) due to the contribution of de-excitations in the former.^98^ As shown below, this possibly leads to different dynamics and also shows up on the time-resolved descriptors.

Then, standard Slater–Condon rules can be used to calculate the dipole matrix elements between such singly excited configurations
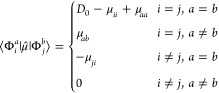
2where  and the generic μ⃗_*st*_ is the dipole moment matrix element in the molecular-orbital (MO) representation.

### II.II Real-Time Propagation

The time-dependent Schrödinger equation is given by (atomic units are used in this work)
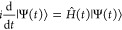
3where |Ψ(*t*)⟩ is the time-dependent wavefunction and *Ĥ*(*t*) is the time-dependent Hamiltonian, which includes the field-free Hamiltonian *Ĥ*_*el*_ and the interaction between the system dipole operator  with the external field *F⃗*(*t*)
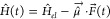
4TDDFT and GW/BSE eigenstates have been labeled by 1, 2, ... to indicate the first, second excited state, etc. For the electronic ground state, we have |0⟩ ≡ |Φ_0_⟩.

The electronic time-dependent wavefunction is expanded over the number *N*_states_ of eigenstates (the DFT ground state plus the *N*_states_ – 1 TDDFT or GW/BSE eigenstates) as
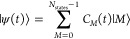
5In the expansion of eq 5, *C*_*M*_(*t*) are time-dependent expansion coefficients, and |*M*⟩ represents the *M*th time-independent TDDFT or GW/BSE eigenstate of the isolated system (eq 1), with an eigenvalue *E*_*M*_.

The matrix form of eq 3 is formally given by
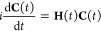
6where **C**(*t*) is the vector of the time-dependent expansion coefficients and **H**(*t*) is the matrix representation at time *t* of *Ĥ*(*t*) on the basis of the TDDFT or GW/BSE eigenstates (**H**(*t*))_*LM*_ = ⟨*L*|*Ĥ*(*t*)|*M*⟩. The coefficients **C**(*t*) are propagated via a second-order Euler algorithm as implemented in the WaveT code.^43−45^ The time-dependent Hamiltonian *Ĥ*(*t*) is written in terms of the eigenenergies *E*_*M*_ and the transition dipole moments ⟨*L*|μ̂|*M*⟩ as
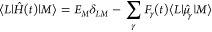
7where γ = *x*, *y*, or *z* indicates the Cartesian component of the dipole and of the field. ⟨*L*|μ̂|*M*⟩ are easily computed from eqs 1 and 2. We have employed a Gaussian envelope function for the time-dependent external field
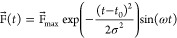
8where F⃗_max_ is the field amplitude (the intensity *I* = 1/2|F⃗_max_|^2^), *t*_0_ and σ are the center and the amplitude of the Gaussian, respectively, and ω is the pulse frequency.

### II.III Time-Dependent Wavepacket-Induced Density

The transition density associated with the *M*th electronic transition is defined in its diagonal form as
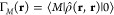
9where ρ̂(**r**) ≡ ρ̂(**r**, **r**) is the reduced one-electron density matrix operator. Γ_*M*_(**r**) is a real quantity extracted by the ADF output.

The derivation of the time-dependent wavepacket-induced density starts from the time-dependent expectation value of ρ̂(**r**):
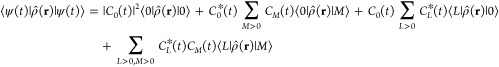
10

In the case of the electron wavepacket, we refer to induced density because a number *M* of different excitations and transition densities Γ_*M*_(**r**) are involved. From eq 10, we can define the time-dependent wavepacket-induced density Γ(**r**, *t*) as
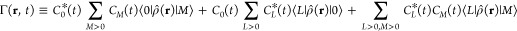
11where *C*_0_^*^(*t*)∑_*M*>0_*C*_*M*_(*t*)⟨0|ρ̂(**r**)|*M*⟩ and *C*_0_(*t*)∑_*L*>0_*C*_L_^*^(*t*)⟨*L*|ρ̂(**r**)|0⟩ are complex conjugates. When only a linear regime is considered, with a low enough external field, we exploit *C*_0_(*t*) ∼ 1 along the whole time propagation. Considering this, and applying *C*_*M*_(*t*) + *C*_M_^*^(*t*) = 2Re[*C*_*M*_(*t*)], one obtains
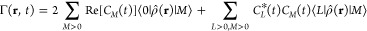
12which can be split into Γ(**r**, *t*) = Γ_1_(**r**, *t*) + Γ_2_(**r**, *t*), with
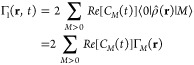
13and
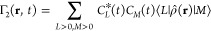
14

Γ_2_(**r**, *t*) can be neglected since a small intensity, which would lead to a very small excited-state population, is assumed. Moreover, only the real part of the time-resolved expansion coefficients should be considered for calculating Γ_1_(**r**, *t*). In this work, Γ_1_(**r**, *t*) is only computed within a linear regime, otherwise, the explicit inclusion of *C*_0_(*t*) and *C*_0_^*^(*t*) must be taken into account.

### II.IV Time-Dependent ΔPDOS

Assuming that the wavepacket at an initial time is the ground state of the system, i.e., Ψ(*t* = 0) = |0⟩, the initial PDOS_ini_(ε) is defined as
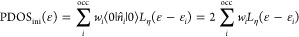
15where *n̂*_*i*_ is the number operator, and *L*_η_(ε – ε_*i*_) is a Lorentzian function centered on MO energies ε_*i*_ and with width η, used to obtain a smooth profile
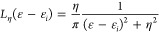
16while *w*_*i*_ is the Mulliken weight for the *i*th MO (see below). Factor 2 results from integration over the spin variable (only closed-shell systems are considered).

If one decomposes the system onto a set of *FF* disjoint fragments (i.e., each atom belongs to only one of the fragments into which the molecule is subdivided), the collective Mulliken weight for MO *i* (occupied or virtual) is
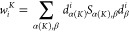
17where α runs over the basis functions centered at atoms belonging to fragment *K* (*K* ∈ {1, 2, ..., *FF*}), β runs over all the basis functions, *S*_α(_*K*),β is the overlap between basis functions, and *d*_α(*K*)_^*i*^ and *d*_β_^*i*^ are the linear coefficients of the MOs expansion in the atomic-orbital (AO) basis set. If only a fragment is defined, i.e., the entire system, the sum over α has no constraints, and one recovers the total Mulliken weight *w*_*i*_.

The time-dependent PDOS_*K*_(*t*,ε) for fragment *K* is defined as the expectation value with respect to |Ψ(*t*)⟩ of the number operator *n̂*_*i*_ weighted by *w*_*i*_^*K*^. We are interested in ΔPDOS_*K*_(*t*,ε) = PDOS_*K*_(*t*,ε) – PDOS_ini_(ε), which is explicitly given by
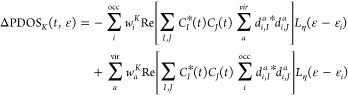
18The sum over *I* and *J* explicitly includes the electronically excited states of the system since the pure ground-state contribution is excluded by construction and cross terms as *C*_I_^*^(*t*)*C*_0_(*t*), deriving from ⟨*I*|*n̂*_*i*_|0⟩, are exactly zero.

### II.V Time-Dependent TCM

TCM descriptor, originally defined in ref^42^ and recently implemented within the PolTDDFT framework,^99−101^ is extended to the time domain by
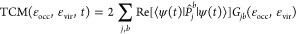
19where the projector *P̂*_*j*_^*b*^ extracts the Slater determinant |Φ_*j*_^*b*^⟩ from the *M*th electronic state
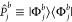
20
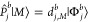
21Using the expansions in eqs 1 and 5, and ⟨*L*|*P̂*_*j*_^*b*^|*M*⟩ = (*d*_*j*,*L*_^*b*^)*d_*j*,*M*_^*b*^, one obtains
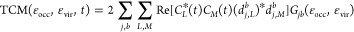
22with
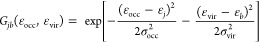
23being a Gaussian used for convolution. Choosing a Gaussian instead of a Lorentzian, as done for the ΔPDOS_*K*_(ε, *t*) is only practical. ε_occ_ and ε_vir_ are the energies of occupied and unoccupied (virtual) MOs, respectively, whereas σ_occ_ and σ_vir_ are the widths of the Gaussian function. The sum over *j* and *b* runs over occupied and virtual MOs, and the sum over *L* and *M* runs over the number of electronically excited states.

Assuming two fragments *K* and *P*, we have also defined the projected time-dependent TCM via a set of Mulliken weights {*w*^*K*^}. Four different TCM maps can therefore be computed for each (*K*,*P*) fragment pair
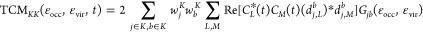
24
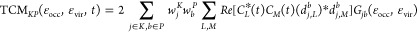
25
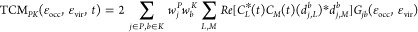
26
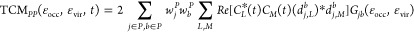
27The four equations describe, respectively, the time evolution of TCM with both sets of MOs projected on fragment *K* (TCM_*KK*_(ε_occ_, ε_vir_, *t*)), with occupied MOs projected on *K* and virtual MOs on *P* (TCM_KP_(ε_occ_,ε_vir_,*t*)) and vice versa (TCM_*PK*_(ε_occ_,ε_vir_,*t*)), and with both sets of MOs projected on fragment *P*. These TCM maps allow one to identify the orbital from which charge departs in the fragment *K* or *P*, and the orbital in other fragments to which charge arrives.

## III Computational Details

The ground-state structure of HBDI has been optimized at the B3LYP/6-31G(d) level using Gaussian,^102^ while ground-state structures of DNQDI and LiCN have been taken from refs^93^ and (44), respectively. The geometry of the Ag_22_ cluster has been obtained by extracting a nanowire from the structure of the fcc bulk gold grown along the 110 direction, as done previously for gold nanowires.^103^ The length of the nanowire has been then reduced by keeping only 22 atoms, changing the Au atoms with Ag ones, and setting the interatomic distance to 2.88 Å.

CAM-B3LYP, B3LYP, and PBE functionals have been chosen for the TDDFT calculations for HBDI, DNQDI, and LiCN, with a TZP basis set of Slater-type orbitals taken from the ADF database. A DZ basis set optimized for ZORA calculations has been chosen for Ag_22_. GW/BSE calculations (in the G_*n*_W_*n*_ variant^47^) were performed using the cc-pVTZ basis for the DNQDI and HBDI molecules, whereas the 6-31G basis set was used for LiCN. Resolution of identity was adopted for the calculation of four-center integrals. For the DNQDI molecule, core states up to the 114th state and virtual states above the 1400th were kept frozen in the calculation of response functions, whereas for HBDI and LiCN all virtual orbitals allowed by the dimension of the basis set were included. GW/BSE calculations were performed on top of the DFT ones employing the PBE0 functional in the case of DNQDI, PBE for HBDI, and CAM-B3LYP for LiCN. The TDA was used for GW/BSE calculations, and TDDFT results were obtained using both the full response and the TDA as implemented in ADF.

**Figure 2:**
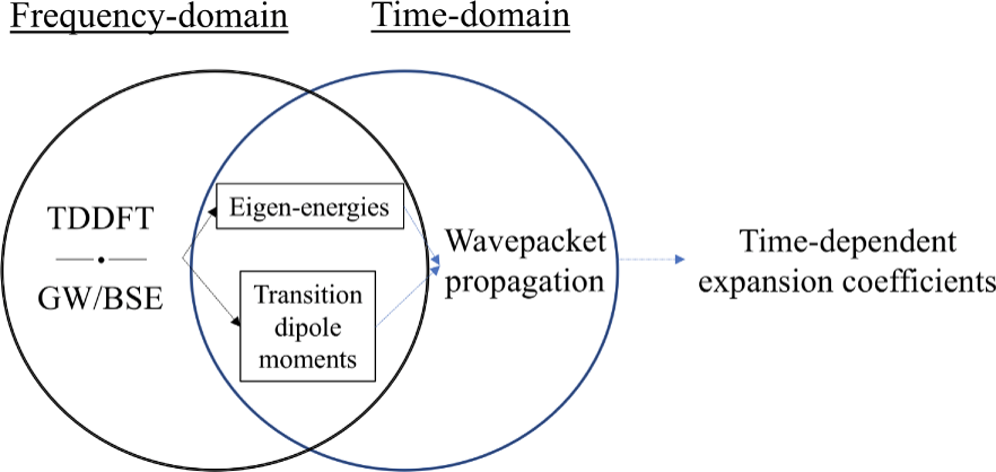
Sketch representation of the implemented interface between electronic structure packages (ADF and MolGW) and the propagation code (WaveT).

Real-time propagations (eq 6) have been performed using the in-house WaveT code,^43^ recently interfaced with both ADF and MolGW. Practically, TDDFT and GW/BSE calculations provide excitation energies and transition dipole moments, which are then used as input parameters for the electronic wavepacket dynamics (eq 7). A schematic representation of the interface between ADF/MolGW and WaveT is shown in Figure 2. A second-order Euler scheme has been used to propagate TDSE, with a time step δ*t* of 0.121 as for HBDI, DNQDI, LiCN, and Ag_22_.

For HBDI and DNQDI, 400 fs dynamics with a pulse intensity of 5 × 10^8^ W/cm^2^ has been carried out. The full width at half-maximum (FWHM) of 15 and 25 fs has been used (FWHM = 2.355σ for a Gaussian envelope, as in eq 8). A delay time Δ*t* of 10 and 70 fs between the two pulses has been employed. Two pulses polarized along the *z* axis (see Figure 1) were applied to HBDI and DNQDI for both levels of theory.

For HBDI, 6 (CAM-B3LYP), 7 (B3LYP), 10 (PBE), and 6 (BSE) electronic excited states have been, respectively, included in the calculations to span an excitation energy range of about 5 eV. The same numbers of excited states have been used for full TDDFT and TDA simulations. For HBDI, at the TDDFT/CAM-B3LYP level, the first pulse has a central frequency of 3.679 eV, which is resonant with the |0⟩ → |1⟩ transition (|1⟩ is the first bright excited state of HBDI). The second pulse is characterized by a central frequency of 1.112 eV, corresponding to the |1⟩ → |4⟩ excitation. The same excitations are selected at the TDDFT/TDA level, with energies equal to 3.961 and 0.924 eV. At the GW/BSE level of theory, the first pulse is resonant with the |0⟩ → |2⟩ transition with a frequency of 3.962 eV. The |2⟩ state is the first bright excited state with BSE. The second pulse is instead characterized by a frequency equal to 0.861 eV, coinciding with the |2⟩ → |4⟩ excitation. B3LYP and PBE excitations are collected in Table S1 of the Supporting Information (SI).

For DNQDI, 50 (CAM-B3LYP), 50 (B3LYP), 100 (PBE), and 60 (BSE) electronic excited states have been, respectively, taken into account for the wavepacket propagation to cover an energy range of around 5 eV. Also for DNQDI, full TDDFT and TDA share the same number of states. For DNQDI, at TDDFT/CAM-B3LYP, the first-pulse frequency is of 1.995 eV, which is resonant with the |0⟩ → |1⟩ excitation. The second pulse has a central frequency equal to 0.718 eV, corresponding to the |1⟩ → |2⟩ transition. At the BSE level, the first-pulse frequency is equal to 2.050 eV, resonant with the |0⟩ → |1⟩ excitation. The second pulse is characterized by a central frequency equal to 0.734 eV, which is equal to the |1⟩ → |3⟩ transition. The same excitations as for BSE are reported for TDDFT/TDA, with energies equal to 2.114 and 0.741 eV, respectively. B3LYP and PBE excitations are collected in Table S1 of the SI.

LiCN dynamics was studied up to 100 fs by a single pulse, with an intensity of 10^3^ W/cm^2^ and an FWHM of 15 fs. The pulse is polarized along the *z* axis. For LiCN, the lowest 10 (CAM-B3LYP, B3LYP, and PBE) and 15 (BSE) excited states were considered. At the TDDFT/CAM-B3LYP level, the |0⟩ → |1⟩ transition centered at a frequency of 5.413 eV, which involves an inversion of the dipole sign, and the |0⟩ → |4⟩ one not presenting this switch in the dipole, with a central frequency of 6.266 eV, were studied. Regarding the GW/BSE calculations, the 4.907 and 8.423 eV transition energies resonant with the |0⟩ → |1⟩ and |0⟩ → |11⟩ excitations, related, respectively, to a dipole switch and not-dipole inversion situations, were evaluated. B3LYP and PBE excitations are reported in Table S1 of the SI. All of the transitions with full TDDFT, TDDFT/TDA (both with CAM-B3LYP), and BSE are reported in Table 1.

**Table 1:** Excitations and Corresponding Energies Studied in the Work for HBDI, DNQDI, LiCN, and Ag_22_^a^

	full TDDFT		TDDFT/TDA		BSE	
**HBDI**	excitation	energy (eV)	excitation	energy (eV)	excitation	energy (eV)
	|0⟩ → |1⟩	3.679	|0⟩ → |1⟩	3.961	|0⟩ → |2⟩	3.962
	|1⟩ → |4⟩	1.112	|1⟩ → |4⟩	0.924	|2⟩ → |4⟩	0.861

	full TDDFT		TDDFT/TDA		BSE	
**DNQDI**	excitation	energy (eV)	excitation	energy (eV)	excitation	energy (eV)
	|0⟩ → |1⟩	1.995	|0⟩ → |1⟩	2.114	|0⟩ → |1⟩	2.050
	|1⟩ → |2⟩	0.718	|1⟩ → |3⟩	0.741	|1⟩ → |3⟩	0.734

	full TDDFT		TDDFT/TDA		BSE	
**LiCN**	excitation	energy (eV)	excitation	energy (eV)	excitation	energy (eV)
	|0⟩ → |1⟩	5.413	|0⟩ → |1⟩	5.430	|0⟩ → |1⟩	4.907
	|0⟩ → |4⟩	6.266	|0⟩ → |4⟩	6.278	|0⟩ → |11⟩	8.423

	full TDDFT		TDDFT/TDA		BSE	
**Ag**_22_	excitation	energy (eV)	excitation	energy (eV)	excitation	energy (eV)
	max abs.	3.476	max abs.	4.533	-	-

a HBDI, DNQDI, and LiCN: CAM-B3LYP functional has been used for “Full TDDFT” and “TDDFT/TDA”. Ag_22_: PBE functional has been used for “Full TDDFT” and “TDDFT/TDA”.

For Ag_22_, TDDFT calculations using both the full response and TDA have been performed, and 500 electronic excited states have been generated at the TDDFT level of theory. The pulse is linearly polarized along the *x* direction, while its frequency has been chosen to approximately coincide with the absorption maximum with the *x*-polarized pulse, i.e., 3.476 eV for full TDDFT and 4.533 eV for TDDFT/TDA. The pulse FWHM is 15 fs. The value of η in eq 16 and the values of σ_occ_ and σ_vir_ in eq 23 are 0.027 eV for HBDI, DNQDI, and LiCN. A value of 2.7 × 10^–3^ eV has been used for Ag_22_eq 23.

Nuclei have been kept frozen at their equilibrium position during the propagation.

## IV Results and Discussion

The present results on HBDI, DNQDI, LiCN, and Ag_22_ show the application of the time-resolved tools described in Section II. HBDI and DNQDI have been investigated by a “pump–probe experiment” with the main goal to describe excited-state populations and to compute excited-state dipoles. LiCN is the well-known prototype of dipole-switch systems: this property is easily analyzed by our time-dependent formulation, as shown below. The collective behavior of Ag_22_ excitations has been investigated by time-resolved TCMs.

We provide here a direct comparison between TDA and full TDDFT results for some of the descriptors, when useful for identifying method-specific time-dependent features.

### IV.I HBDI and DNQDI

The expectation value of the dipole of the first bright excited state has been computed for HBDI and DNQDI and is reported in Table 2, at TDDFT, TDA, and BSE levels of theory. For TDDFT and TDA, we tested the CAM-B3LYP, B3LYP, and PBE functionals, while BSE simulations have been performed only within TDA. For HBDI, both TDDFT and BSE estimates compare well the reference ab initio result of 5.16 D, obtained by means of SAC-CI. This comparison indicates that the formalism shown in Section is appropriate for getting excited-state properties when double excitations are absent or negligible. At the full TDDFT level, B3LYP and PBE overestimate the excited-state dipole, while TDA produces larger values of the dipole with respect to the full-TDDFT: the difference between TDDFT and TDA results is the largest for PBE and the smallest for CAM-B3LYP. To the best of our knowledge, the only theoretical reference for DNQDI is provided by some of us at the B3LYP level, in the recent work.^93^ Estimates of DNQDI excited-state dipole are all close to zero. A space representation of the excited dipole for HBDI is given in Figure S1 of the SI.

**Table 2:** Dipole (in Debye) of the first bright excited state for HBDI and DNQDI

	CAM-B3LYP	B3LYP	PBE	CAM-B3LYP/TDA	B3LYP/TDA	PBE/TDA	BSE	Lit.
HBDI	5.46	5.92	6.40	5.59	6.32	7.51	5.06	5.16^a^
DNQDI	0.19	0.02	0.04	0.02	0.03	0.07	0.02	0.00^b^

a SAC-CI in ref (82).

b B3LYP in ref (93).

**Figure 3:**
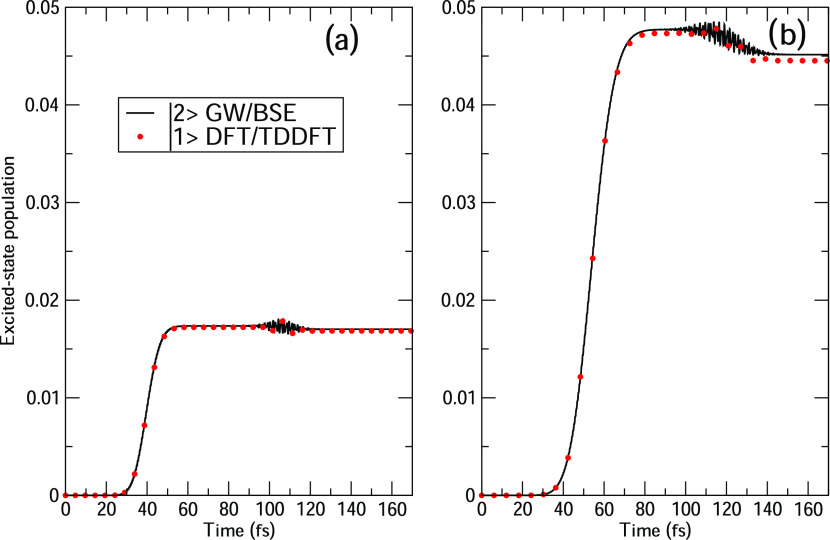
Time-evolution of HBDI excited-state populations computed at TDDFT/TDA and BSE levels, with a delay time Δ*t* of 70 fs between the two pulses: (a) FWHM = 15 fs and (b) FWHM = 25 fs. The CAM-B3LYP functional has been used for TDDFT/TDA.

**Figure 4:**
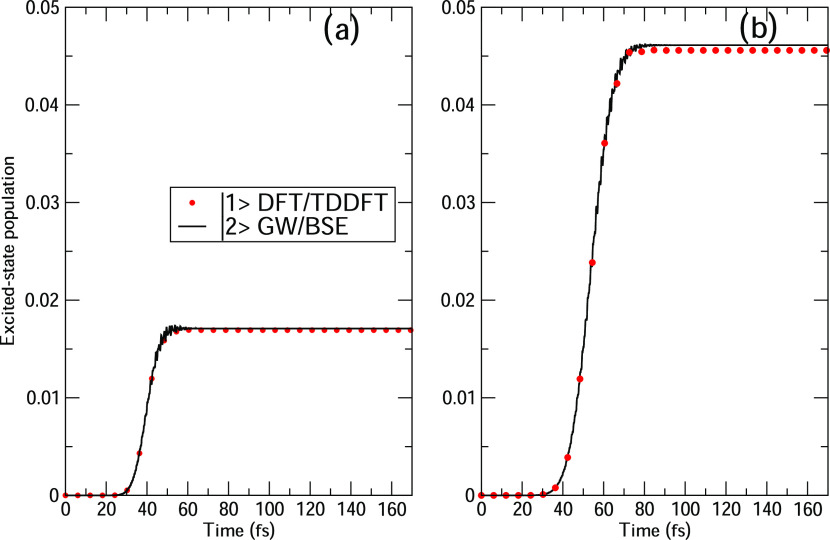
Time-evolution of HBDI excited-state populations computed at TDDFT/TDA and BSE levels, with a delay time Δ*t* of 10 fs between the two pulses: (a) FWHM = 15 fs and (b) FWHM = 25 fs. The CAM-B3LYP functional has been used for TDDFT/TDA.

To check the implementations outlined in Section II about transition dipole moments between excited states, we have simulated pump–probe experiments for HBDI and DNQDI to study the electron excited-state dynamics in the presence of two pulses, separated by a delay time Δ*t* of 10 and 70 fs. The first pulse is resonant with the transition from the ground state to the first bright state of the molecule, while the frequency of the second pulse has been chosen to be resonant with the transition from the first bright state to a higher state. Since the applied intensity is small enough (see Section III), we expect that the population of higher-energy states is only a small fraction of that of the first bright state. We have used both a DFT/TDDFT and a GW/BSE wavepacket in the dynamics. We have computed populations for HBDI and DNQDI with two values of pulse FWHM (15 and 25 fs), which are reported in Figures 3 and 4 for HBDI.

For HBDI, at the TDDFT/CAM-B3LYP level, the first bright state is |1⟩, while it is |2⟩ at the GW/BSE level. In both cases, TDA has been applied for a meaningful comparison. Excited states are characterized by a strong transition dipole moment along the *z-*axis, with a highest occupied molecular orbital–lowest unoccupied molecular orbital (HOMO–LUMO) transition. Moreover, the “probe” is resonant with a transition from the first bright state to |4⟩ along the *z-*axis.

In Figure 3, we report the time evolution of the first bright excited state of HBDI with a delay time of 70 fs between the two pulses. The left (right) panel of Figure 3 contains results with FWHM = 15 fs (25 fs). The value of the delay time Δ*t* is large enough to exclude any superposition of the two pulses, i.e., state populations are not affected by any interference between the two pulses. A longer pulse duration (right panel of Figure 3) generates higher state populations, as expected. The first pulse terminates approximately at 110 fs, beyond which the |1⟩ TDDFT or |2⟩ BSE population decreases because of the effect of the second pulse. Since the applied intensity is low (see Section III), only a linear response is obtained, with small values of the various populations involved in the dynamics. TDDFT/CAM-B3LYP and BSE populations are substantially superimposed at any time and for any FWHM value.

Generally, the same comments may be extended to the case of a shorter delay time, i.e., 10 fs (Figure 4). One major exception is however found: the time evolution of the |1⟩ (|2⟩) population is not characterized anymore by a step decrease because the second pulse now partially overlaps with the first one. This does not substantially change the asymptotic value of the populations of the first bright state when compared with the values in Figure 3.

The effect of removing TDA and of using different functionals is shown in the SI. In particular, full-TDDFT populations are smaller than the TDDFT/TDA ones, as shown in Figure S2 of the SI, where the representative case with Δ*t* = 10 fs and FWHM = 15 fs is reported. A comparison between CAM-B3LYP, B3LYP, and PBE results, obtained using full TDDFT, is reported in Figures S3 and S4 of the SI: CAM-B3LYP and B3LYP populations are close to each other, while the PBE ones show smaller asymptotic values for any delay time and FWHM.

Excited-state populations of DNQDI are collected in Figures S5 and S6 of the SI, using TDDFT/TDA and BSE. Also for DNQDI the two pulses are linearly polarized along the *z-*axis. The first bright excited state is |1⟩ in both TDDFT and BSE simulations. The |0⟩ → |1⟩ is strongly dominated by the HOMO → LUMO transition. As for HBDI, the second pulse has been chosen to be resonant with an excited-excited transition along the *z-*axis: the final state is |2⟩ for TDDFT/TDA/CAM-B3LYP and |3⟩ for BSE. By increasing the pulse duration, the population of excited states visibly increases, with BSE providing larger asymptotic populations than TDDFT, at variance with what occurs for HBDI. Also for DNQDI, TDDFT/TDA gives larger populations than full TDDFT for all the functionals employed, as shown in Figure S7 of the SI for Δ*t* = 10 fs and FWHM = 15 fs: CAM-B3LYP produces the largest population, with PBE giving the smallest one, as already observed for HBDI.

As a final comment, we point out that the extension of the transition dipole moment of excited–excited pairs, as reported in Section II, has been first tested by comparing with the excited-state dipoles reported in Table 2, showing a good agreement with the literature theoretical data. Second, we have applied this formalism to *ad hoc* “pump–probe experiments" to investigate the behavior of the electronic wavepacket when interrogated by excited–excited resonant pulses.

**Figure 5:**
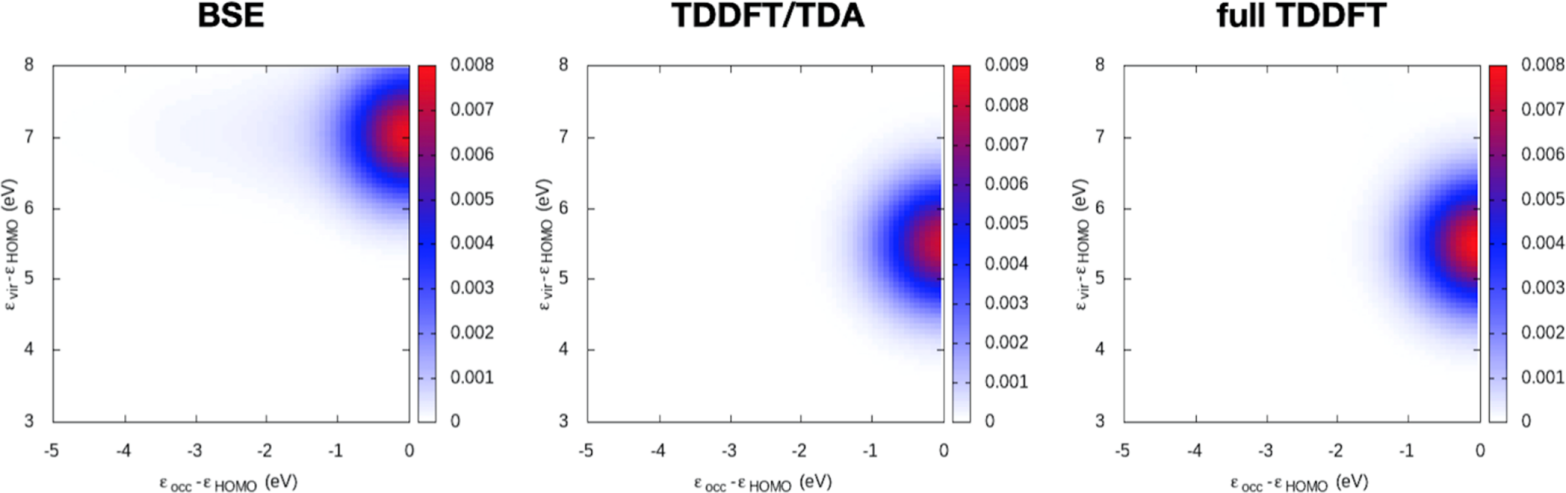
HBDI TCM at 36 fs, with a delay time Δ*t* of 10 fs and FWHM = 15 fs. Left: BSE; center: TDDFT/TDA with CAM-B3LYP; right: full TDDFT with CAM-B3LYP.

As a representative example for TCM analysis (eq 22), we report in Figure 5 the TCM for HBDI, computed at around 36 fs, i.e., at the maximum field amplitude for the first pulse, and FWHM = 15 fs. Figure 5 contains three panels with the TDDFT, TDDFT/TDA, and BSE results: MO energy values are Kohn–Sham (KS) and GW. As already pointed out, the first pulse triggers a pure HOMO → LUMO transition in HBDI. As a consequence, all of the TCM maps in Figure 5 show a single spot: for HBDI, the KS HOMO–LUMO gap is smaller than the corresponding GW one. The same comment may be extended to DNQDI TCMs, which are however not reported here.

**Figure 6:**
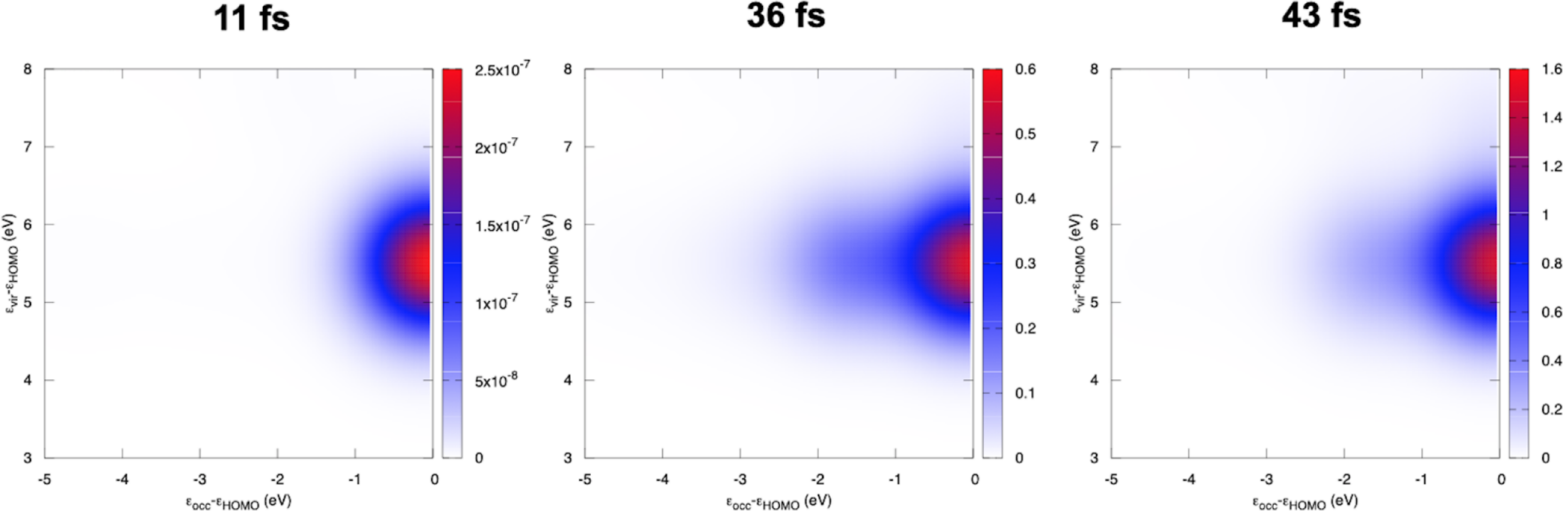
Time-resolved TCM of HBDI at full TDDFT/CAM-B3LYP level, at 11, 36, and 43 fs. Note that the color scale is different in the three panels. Intensity = 5 × 10^12^ W/cm^2^.

TCMs for HBDI, calculated for both B3LYP and PBE functionals at full TDDFT and TDA levels, are reported in Figure S8 of the SI at 36 fs. To observe time-dependent changes in TCM, we applied a higher intensity of 5 × 10^12^ W/cm^2^ to the two-pulse HBDI case, with a delay time of 10 fs and FWHM of 15 fs. The nonlinear HBDI response involves inner orbitals and a substantial population of the excited states under consideration. In Figure 6, we report the TCM at 11 fs (first pulse increase), 36 fs (maximum of the first pulse), and 43 fs (maximum of the second pulse), computed at TDDFT/CAM-B3LYP. We observe that the shape of the spot changes in time, other than the intensity of the signal. The 11 fs TCM coincides with that in the upper panel of Figure 5, which has been obtained with an intensity equal to 5 × 10^8^ W/cm^2^: only the first pulse is present, with an amplitude enough to only determine the linear response, i.e., the HOMO–LUMO transition. At 36 and 43 fs, the TCM spot shows a shoulder corresponding to transitions involving the inner occupied orbitals. The energy difference between HOMO and HOMO–2 and HOMO–3 is around 1.7 eV, consistent with the signal observed at negative energies. We can assume that depopulating HOMO–2 and HOMO–3 originates from |1⟩ → |4⟩ or, more generally, from the combination of the two pulses in the nonlinear regime. Indeed, the same calculation with only the first pulse does not give the shoulder in TCM at 36 and 43 fs. This feature is also confirmed by inspecting the time evolution of the involved MO occupation numbers, reported in Figure S9 of the SI; HOMO–1 and MOs higher than LUMO do not show an appreciable occupation under these conditions. The asymptotic populations of |1⟩ and |4⟩ states are in this case 0.44 and 0.34: when compared with the values in the linear regime (left panel of Figure 4), an increase of around one order of magnitude is found for |1⟩, and of three orders of magnitude for |4⟩. This finding confirms that the second excited state involved by the second pulse is largely populated. Other time-dependent features are observed, as reported in Figure S10 of the SI: we observe that the HOMO–2/HOMO–3→ LUMO transition can be dominant (54 fs), and that higher virtual orbitals are also involved (58 fs). These features repeat at larger times, together with that at 36 and 43 fs, giving evidence of the time evolution of a superposition of different excited states.

**Figure 7:**
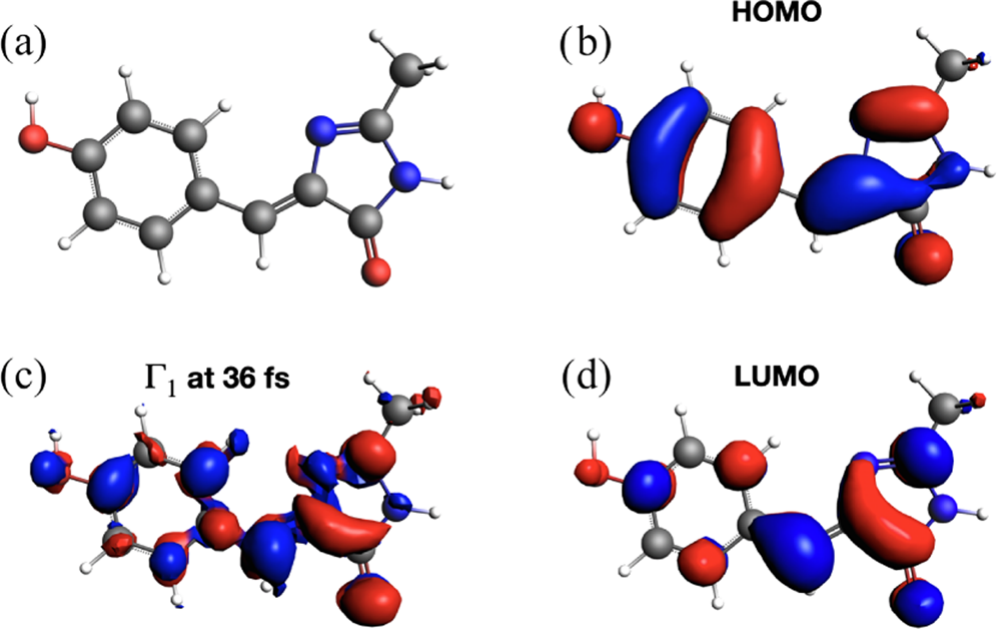
Induced density of HBDI (a). The calculated time-resolved induced density (c) is shown, compared with HOMO (b) and LUMO (d). Snapshot at 36 fs.

**Figure 8:**
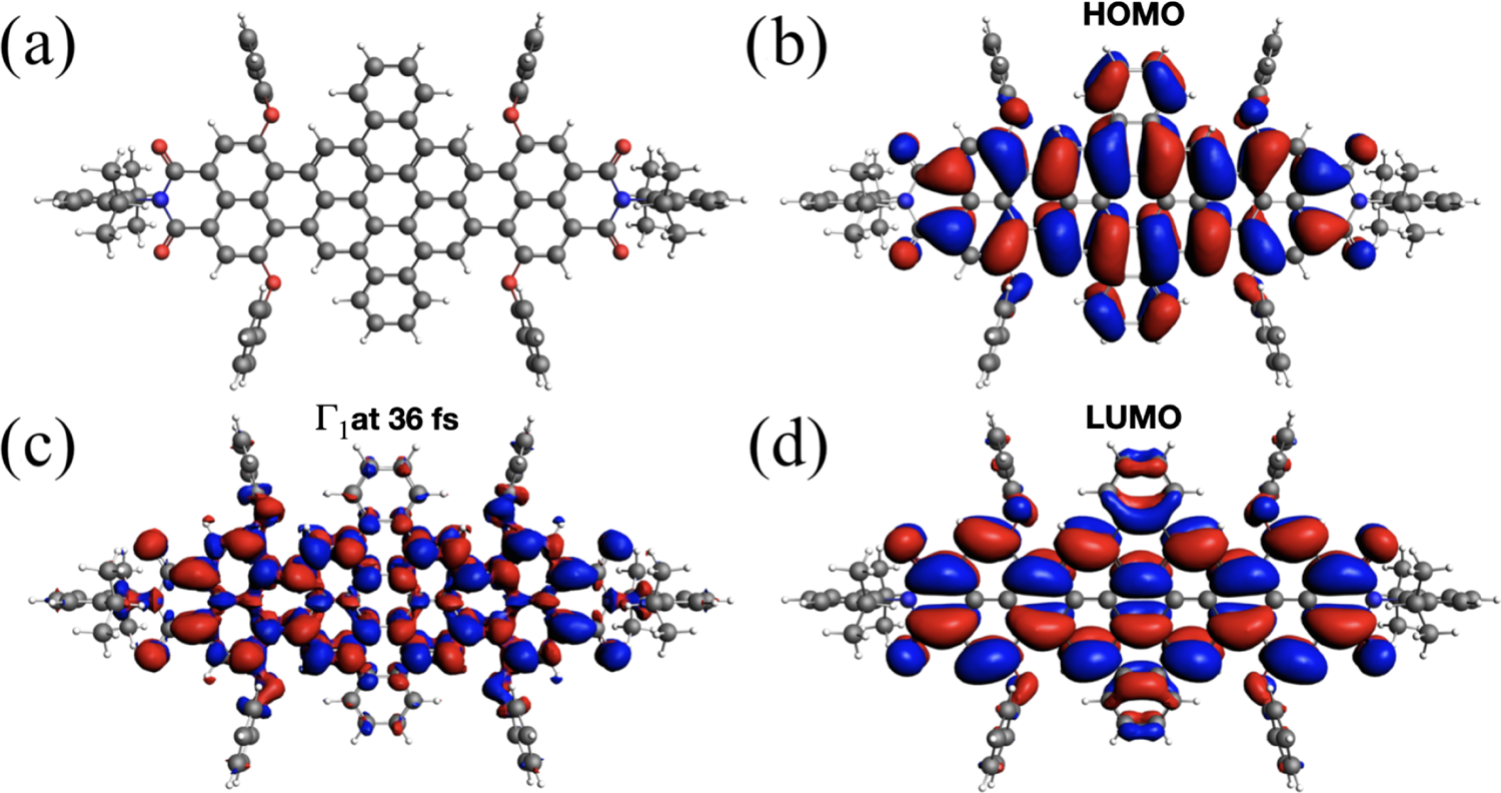
Induced density of DNQDI (a). The calculated time-resolved induced density (c) is shown, compared with HOMO (b) and LUMO (d). Snapshot at 36 fs.

Real-time TDDFT trajectories of HBDI and DNQDI have been also analyzed using the time-dependent induced density Γ_1_ in eq 13. Snapshots at 36 fs are reported in Figures 7 and 8, together with the HOMO and LUMO spatial representations. For HBDI in Figure 7, one observes that spatial features of Γ_1_ (panel c) closely resemble those of LUMO (panel d), as expected. The analysis of Γ_1_ for DNQDI (Figure 8) shows a complex nodal structure of the induced density. In both cases, the computed induced density is a combination of HOMO and LUMO.

These features are somewhat expected and indeed the aim of the applications to HBDI and DNQDI reported in the present work is to show that such kind of time-resolved analysis is affordable at TDDFT and BSE levels for large systems.

### IV.II LiCN

LiCN has been extensively used as a model molecule to study electron dynamics, intramolecular charge transfer, and photoionization.^21,104,105^ DFT/TDDFT and GW/BSE wavepackets have been propagated under the influence of an external pulse to study intramolecular charge-transfer features. For the analysis of the propagation, we have made use of the time-resolved induced density, also used for HBDI and DNQDI (see above), and the time-resolved ΔPDOS, eq 18.

**Figure 9:**
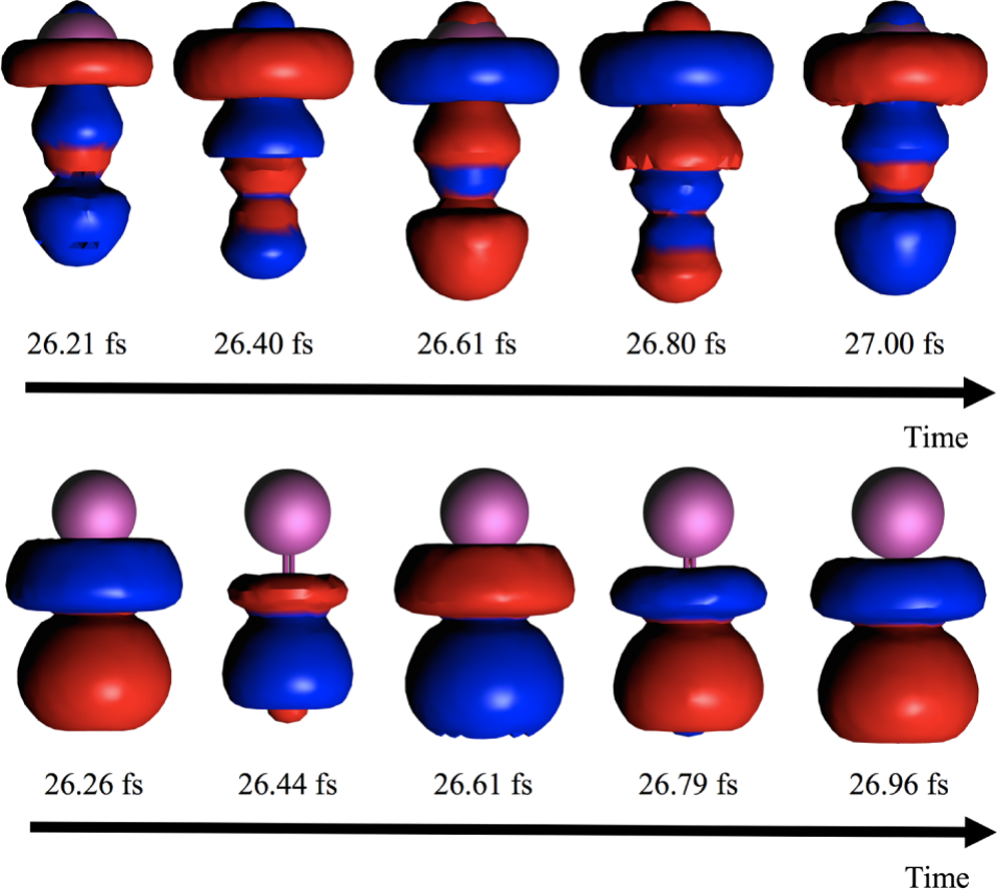
Time-resolved induced density of LiCN at TDDFT/CAM-B3LYP levels, resulting from the application of a pulse resonant with the (a) |0⟩ → |1⟩ (upper panel) and (b) |0⟩ → |4⟩ (lower panel) electronic transitions. Li is in purple.

In Figure 9 we report the time evolution of the induced density in eq 13 around the pulse maximum (26.6 fs) obtained at CAM-B3LYP levels for two pulse frequencies: one resonant with the |0⟩ → |1⟩ transition and the second corresponding to |0⟩ → |4⟩. Both excitations are along the molecular axis: the first transition implies a dipole switch, while the second retains the same dipole direction. The time-resolved induced densities Γ_1_ nicely show the different electron dynamics induced by the two pulses. Density is clearly seen on the Li atom when the dipole switch is activated by the proper pulse (upper panel), whereas the excitation does not involve an arrangement of the electronic density on lithium when the other pulse, resonant with |0⟩ → |4⟩, is employed (lower panel). Plots of the involved orbitals (HOMO and LUMO for |0⟩ → |1⟩, HOMO and LUMO + 1 for |0⟩ → |4⟩) are collected in Figure S11 of the SI.

**Figure 10:**
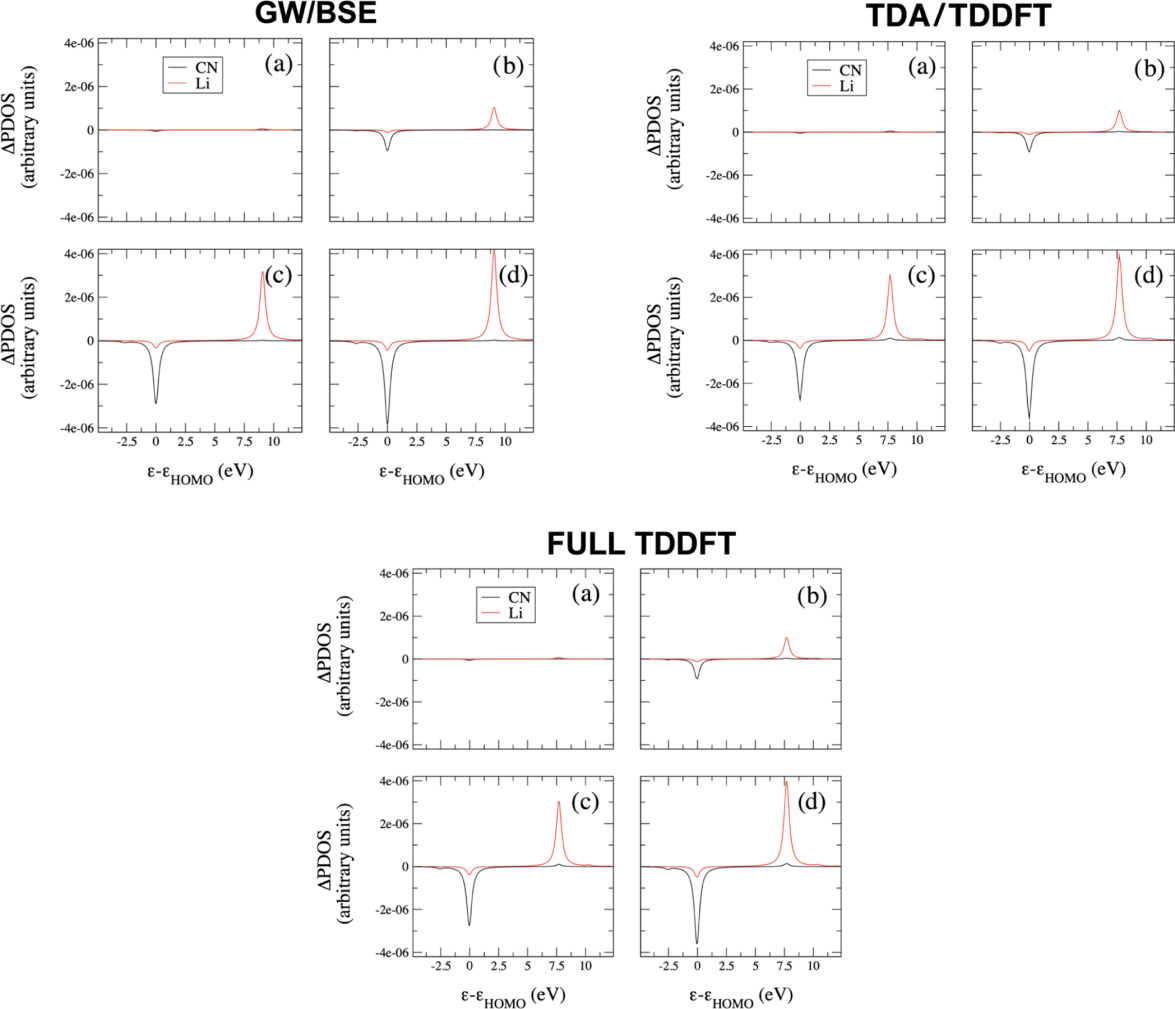
ΔPDOS projected on Li and CN fragments of LiCN for the |0⟩ → |1⟩ transition at (a) 19.4 fs, (b) 26.6 fs, (c) 33.9 fs, and (d) 48.4 fs. Left top panels: BSE; right top panels: TDDFT/TDA using CAM-B3LYP. Lower panels: full TDDFT using CAM-B3LYP.

The ΔPDOS descriptor is particularly useful to analyze the time evolution of charge transfers occurring within a molecular or complex system. ΔPDOS can quantitatively support the information extracted from the time-resolved induced density. We report in Figure 10 four different snapshots (19.4, 26.6, 33.9 and 48.4 fs) of ΔPDOS projected on Li and CN, at DFT/TDDFT GW/BSE (upper panel, left), TDDFT/TDA (upper panel, right) and full TDDFT (lower panel). In this case, only the |0⟩ → |1⟩ has been taken into account, which is the HOMO → LUMO transition, with also HOMO–1 involved. The pulse is switched on at approximately 4 fs; it reaches its maximum near 26.6 fs and it is switched off at around 48.8 fs. Thus, the second and the fourth snapshots correspond approximately to the pulse maximum and termination, respectively. For all the approaches, the projected ΔPDOS on Li and CN clearly indicate a net intramolecular charge transfer, with a net positive (negative) change of electron density on Li (CN), with respect to the initial conditions, i.e., the electronic ground state. Indeed, LUMO is populated, and, at the same time, the HOMO population decreases. We can also observe the timescale at which this pulse-triggered transfer occurs. After 19 fs, the process is still not initiated. At pulse maximum (panel b), the charge transfer is far from its asymptotic value.

The effect of the choice of the functional on ΔPDOS is shown in the left panel of Figure S12 of the SI for the snapshot at 48.4 fs: the main differences are in the Li peak position (reflecting the HOMO–LUMO gap) and in the peak intensities for Li and CN fragments. Changing the functional does not affect the description of the charge transfer but only the position of ΔPDOS peaks. Comparison between full TDDFT and TDA is reported in the right panel of Figure S13 of the SI, for the B3LYP functional only: full TDDFT and TDA produce the same LiCN response. Comparison between the DFT/TDDFT (full and TDA) and the GW/BSE ΔPDOS at 48.4 fs, reported in Figure S13 of the SI, shows that the GW/BSE positive peak on Li is seen to be slightly higher than the corresponding DFT/TDDFT ones. The same behavior is found for the negative peak on CN. Using DFT/TDDFT (full and TDA), the ΔPDOS projected on CN shows a very small positive peak in correspondence with the HOMO–LUMO gap; this feature is instead absent in the GW/BSE ΔPDOS projected on CN. As already pointed out, full TDDFT and TDA results are substantially identical for LiCN.

By integrating the energy in ΔPDOS, one obtains the time evolution of the net change of the charge on the selected fragment. The integrated ΔPDOS on CN and Li fragments is shown in Figure S14 of the SI for TDDFT/CAM-B3LYP and BSE calculations. The electron charge increases in time on Li and, correspondingly, decreases on CN: the two curves are symmetric, as expected. The magnitude of the asymptotic value, which is reached between 45 and 50 fs, depends on the applied pulse intensity. BSE produces a large charge separation than TDDFT/CAM-B3LYP, while full TDDFT and TDDFT/TDA results are substantially superimposed on the scale of the figure. The integrated ΔPDOS as a function of time represents the time variation of the Mulliken charge on a given fragment. A comparison between TDDFT/CAM-B3LYP and BSE results has been also carried out in Figure S15 of the SI, where the time evolution of the minimum and maximum of Li and CN ΔPDOS has been reported. BSE produces larger absolute values than TDDFT, as previously observed for the energy-integrated ΔPDOS; at the same time, BSE curves show a slower decrease/increase rate in the 20–40 fs interval. Full TDDFT and TDDFT/TDA show the same overall behavior, whereas BSE gives large absolute asymptotic values.

**Figure 11:**
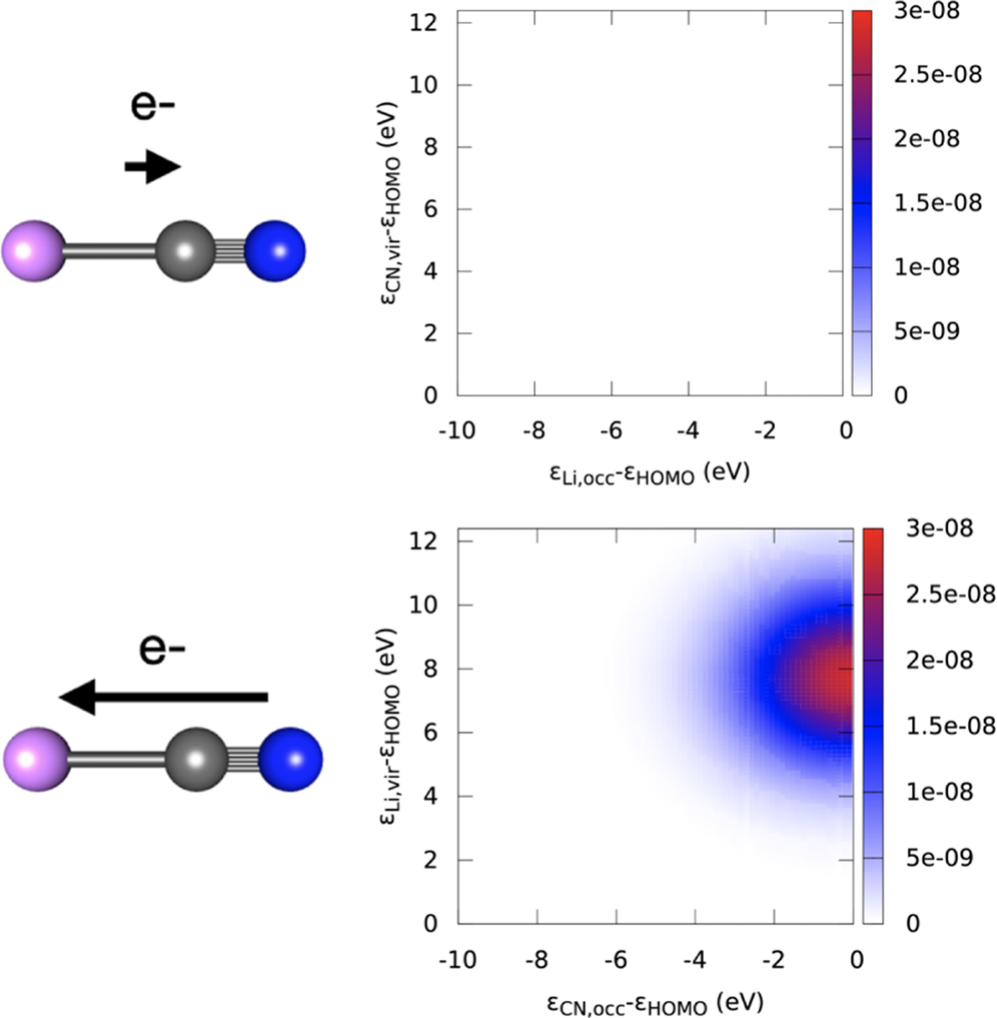
LiCN projected-fragment TCM calculated for the |0⟩ → |1⟩ transition at the TDDFT/CAM-B3LYP level. Upper: Li_occ_ → CN_vir_; lower: CN_occ_ → Li_vir_. Snapshots at 26.61 fs.

Charge-transfer dynamics is also analyzed by means of the projected-fragment TCM_*KP*_(ε_occ_, ε_vir_, *t*), which describes the time-evolution charge-transfer process induced by the external pulse. We report in Figure 11 the projected-fragment TCM for the CN → Li electron transfer (upper panel) and for the opposite process, i.e., the Li → CN when the |0⟩ → |1⟩ pulse is considered at the TDDFT/CAM-B3LYP level of theory. The plotted data refer to 26.1 fs. Only a spot centered at the HOMO–LUMO is observed for CN → Li, as expected by the inspection of the ΔPDOS. The magnitude of the projected-fragment TCM is two orders of magnitude larger than the corresponding Li → CN, and consequently, is not observed using the scale of Figure 11, which is further confirmation that in the simulated conditions a net charge transfer occurs. Indeed, in the representative snapshot described in Figure 11. we observe an electron transition from the HOMO projected on the CN fragment toward the LUMO on the Li fragment; whereas, at the same signal scale, the opposite contribution, i.e., the electron transition from the HOMO projected on Li toward the LUMO on CN, is negligible.

### IV.III Ag_22_

**Figure 12:**
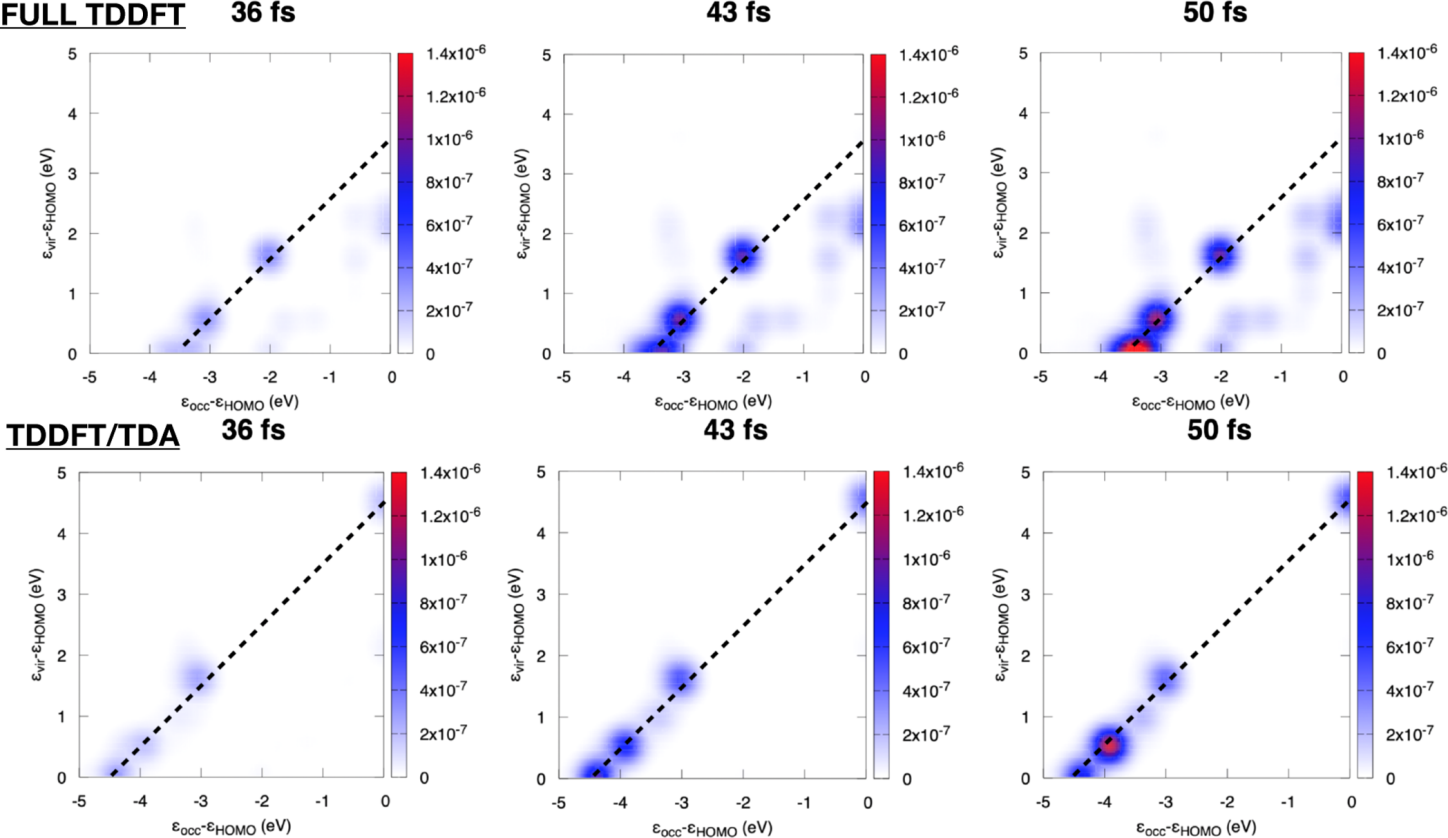
TCM at selected snapshots for Ag_22_ at TDDFT/PBE (upper panels) and TDDFT/TDA/PBE (lower panels) levels of theory. Dashed lines correspond to pulse frequency, 3.476 eV for full TDDFT, and 4.533 eV for TDDFT/TDA.

Other than for molecular systems, the time-resolved descriptors developed in this work may be successfully applied to metal clusters of various sizes. In particular, TCM off-diagonal spots, i.e., not lying along the straight line provided by the frequency of the incident pulse, are evidence of collective behavior in the electronic density response.^96^ Ag_22_ is likely too small to show full plasmonic features, but it can be considered as a simple and small prototype for this kind of analysis. We have applied the time-resolved TCM to Ag_22_, with a pulse linearly polarized along *x* (see Figure 1). The wavepacket has been generated at both full TDDFT and TDDFT/TDA levels of theory using the PBE functional. In Figure 12, snapshots at 36, 43, and 50 fs for TDDFT/PBE and TDDFT/TDA/PBE are reported. We clearly observe off-diagonal spots for the full TDDFT dynamics (upper panels), which indicate a collective contribution to the electronic excitation, while TDA only produces diagonal spots (lower panels). Indeed, the strong impact of TDA on plasmonic modes in finite systems has been already reported in the literature.^106^ In both cases, the intensity of diagonal and off-diagonal spots generally increases with time. Strong diagonal peaks are observed at 50 fs, well beyond the maximum of the pulse (36 fs), indicating a somehow delayed response of the system. Only for full TDDFT, we also observe dynamics of off-diagonal spots, with increased intensity from 36 to 50 fs.

## V Conclusions

We have presented a time-dependent formulation of the induced density, the ΔPDOS and TCM maps, combined with DFT/TDDFT and GW/BSE representations of the electronic ground and excited states of molecular systems, and real-time propagation of the corresponding wavepacket. The time-domain formulation of ab initio descriptors provided here can be considered as a step further for theoreticians to simulate and interpret time-resolved fast and ultrafast experiments.

We have applied our analysis to HBDI, DNQDI, and LiCN molecules, and to the Ag_22_ cluster. In the case of DNQDI, we show how full-electron dynamics on a large molecule can be successfully carried out at the BSE level, i.e., with a highly accurate electronic structure treatment.

Excited-state dipoles for HBDI and DNQDI at the CAM-B3LYP and BSE levels of theory agree well with the literature data. When a nonlinear regime is analyzed for HBDI, the time evolution of TCM shows that the inner occupied orbitals are involved in the electron dynamics.

As a theoretical model for intramolecular charge transfer, LiCN dynamics has been investigated by means of time-resolved induced density, ΔPDOS and energy-integrated ΔPDOS, TCM, and fragment-projected TCM.

Time-resolved descriptors are able to catch differences due to functionals and TDA, and between GW/BSE and TDDFT.

Ag_22_ has been chosen as a simple prototype of systems characterized by the collective electronic response to an external electromagnetic field. Though studying the nature of the optical response of Ag_22_ in terms of possible plasmonic behavior is beyond the scope of this work, we have verified the presence of time-dependent off-diagonal spots in TCMs, usually associated with collective excitations. Off-diagonal spots do not appear when TDA is applied.

Future applications cover a wide range of experiments, from high-harmonic generation in molecules^107^ to plasmon-enhanced photocatalysis. Indeed, as representative examples of time-resolved physical features accessed by our approach, we mention: ionization channels in high-harmonic generation (we will be able to observe at which time ionization from a given MO starts to occur, and possibly measure the delay time between ionization from different MOs); plasmon decay affected by environmental effects, leading to decoherence of the electronic wavepacket.^35,36,38,39,108,109^ A recent interest is indeed rapidly growing about the role of hot carriers in plasmonic-induced catalytic pathways.^110−112^ Our tools will help us to understand the generation and the dynamics of hot carriers, i.e., electrons and holes, in metallic plasmonic clusters and nanoparticles, and their possible injection into close molecules.^36,113^ The computation of the time evolution of the induced density and of ΔPDOS will be therefore easily coupled to a time-resolved multiscale hybrid modeling of a molecule+nanoparticle system.^43,45^

Finally, the postprocessing tools described here can be coupled to the stochastic Schrödinger equation (SSE) framework, with which we can study decoherence effects during the propagation of the wavepacket.^44,45,114^ Studying the influence of electron dephasing or relaxation channels, included in SSE simulations, on the time evolution of the induced density of ΔPDOS and TCM will be therefore accessible for molecular and complex systems.

## Supporting Information Available

Excitation energies at B3LYP and PBE level (full TDDFT and TDA) for HBDI, DNQDI and LiCN; graphical representation of the excited dipole for HBDI; HBDI and DNQDI populations as a function of the functional (CAM-B3LYP, B3LYP and PBE) and of the applied level of theory (full TDDFT or TDDFT/TDA); comparison of TDDFT HBDI time-resolved TCMs for B3LYP and PBE functionals; time-evolution of HBDI HOMO – 3, HOMO – 2, HOMO and LUMO populations at 5 × 10^12^ W/cm^2^; HBDI TCM for nonlinear response at 5 × 10^12^ W/cm^2^; LiCN HOMO, LUMO and LUMO + 1 at CAM-B3LYP level; comparison between DFT/TDDFT and GW/BSE fragmented ΔPDOS for LiCN; energy-integrated ΔPDOS and time evolution of maximum/minimum of ΔPDOS for LiCN (PDF)
tcm_movie_hbdi_fig8 (AVI)
tcm_camb3lyp_0to1_LitoCN.avi (AVI)
tcm_camb3lyp_0to1_CNtoLi.avi (AVI)
induced_density_transfer_LiCN_0to1 (AVI)
induced_density_no_transfer_LiCN_0to4 (AVI)
hbdi_induced_density (AVI)
delta_pdos_camb3lyp_transfer_LiCN_0to1(AVI)
delta_pdos_camb3lyp_no_transfer_LiCN_0to4 (AVI)
delta_pdos_bse_transfer_LiCN_0to1 (AVI)

## Supplementary Material

ct1c00211_si_001.pdf

ct1c00211_si_002.avi

ct1c00211_si_003.avi

ct1c00211_si_004.avi

ct1c00211_si_005.avi

ct1c00211_si_006.avi

ct1c00211_si_007.avi

ct1c00211_si_008.avi

ct1c00211_si_009.avi

ct1c00211_si_010.avi
