# Supplementary material for: Time-Resolved Excited-State Analysis of Molecular Electron Dynamics by TDDFT and Bethe–Salpeter Equation Formalisms
Source: J Chem Theory Comput. 2021 Sep 6;17(10):6314–29. doi: 10.1021/acs.jctc.1c00211 (PMC8515806; doi:10.1021/acs.jctc.1c00211)
Supplement: Supplementary file 1 — ct1c00211_si_001.pdf [file ct1c00211_si_001.pdf]

Supporting Information for "Time-resolved excited-state analysis of molecular electron dynamics by TDDFT and Bethe-Salpeter equation formalisms"

P. Grobas Illobre,[1,2] M. Marsili,[3,a)] S. Corni,[3,4] M. Stener,[1] D. Toffoli,[1,b)] and E. Coccia[1,c)]

[1)]*Dipartimento di Scienze Chimiche e Farmaceutiche, Università di Trieste, via L. Giorgieri 1, Trieste, Italy*

[2)]*Present Address: Scuola Normale Superiore, Piazza dei Cavalieri 7, Pisa 56126, Italy*

[3)]*Dipartimento di Scienze Chimiche, Università di Padova, via Marzolo 1, Padova, Italy*

[4)]*CNR Istituto di Nanoscienze, via Campi 213/A, Modena, Italy*

(Dated: 8 July 2021)

	Full TDDFT/B3LYP		Full TDDFT/PBE		TDDFT/TDA/B3LYP		TDDFT/TDA/PBE	
**HBDI**	Excitation	Energy (eV)	Excitation	Energy (eV)	Excitation	Energy (eV)	Excitation	Energy (eV)
	$\|0\rangle \rightarrow \|1\rangle$	3.524	$\|0\rangle \rightarrow \|2\rangle$	3.273	$\|0\rangle \rightarrow \|2\rangle$	3.839	$\|0\rangle \rightarrow \|2\rangle$	3.627
	$\|1\rangle \rightarrow \|4\rangle$	0.870	$\|2\rangle \rightarrow \|4\rangle$	0.575	$\|2\rangle \rightarrow \|4\rangle$	0.623	$\|2\rangle \rightarrow \|4\rangle$	0.266
	Full TDDFT/B3LYP		Full TDDFT/PBE		TDDFT/TDA/B3LYP		TDDFT/TDA/PBE	
**DNQDI**	Excitation	Energy (eV)	Excitation	Energy (eV)	Excitation	Energy (eV)	Excitation	Energy (eV)
	$\|0\rangle \rightarrow \|1\rangle$	1.730	$\|0\rangle \rightarrow \|1\rangle$	1.475	$\|0\rangle \rightarrow \|1\rangle$	1.863	$\|0\rangle \rightarrow \|1\rangle$	1.632
	$\|1\rangle \rightarrow \|2\rangle$	0.491	$\|1\rangle \rightarrow \|2\rangle$	0.225	$\|1\rangle \rightarrow \|2\rangle$	0.389	$\|1\rangle \rightarrow \|2\rangle$	0.081
	Full TDDFT/B3LYP		Full TDDFT/PBE		TDDFT/TDA/B3LYP		TDDFT/TDA/PBE	
**LiCN**	Excitation	Energy (eV)	Excitation	Energy (eV)	Excitation	Energy (eV)	Excitation	Energy (eV)
	$\|0\rangle \rightarrow \|1\rangle$	4.973	$\|0\rangle \rightarrow \|1\rangle$	4.341	$\|0\rangle \rightarrow \|1\rangle$	4.988	$\|0\rangle \rightarrow \|1\rangle$	4.356

TABLE S1. Excitations and corresponding energies studied in this work for HBDI, DNQDI and LiCN. B3LYP and PBE functionals have been used for "Full TDDFT" and "TDDFT/TDA".

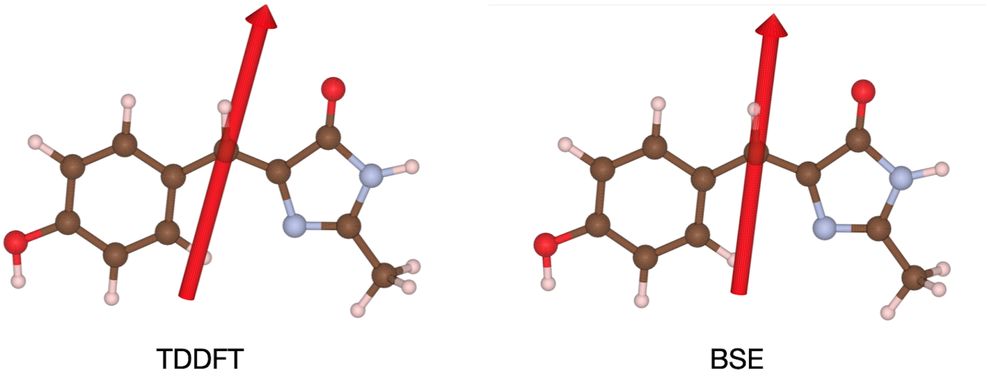

FIG. S1. Expectation value of the dipole of the first bright excited state for HBDI, at TDDFT/CAM-B3LYP and BSE level.

[a)]Electronic mail: margherita.marsili@unipd.it
[b)]Electronic mail: toffoli@units.it
[c)]Electronic mail: ecoccia@units.it

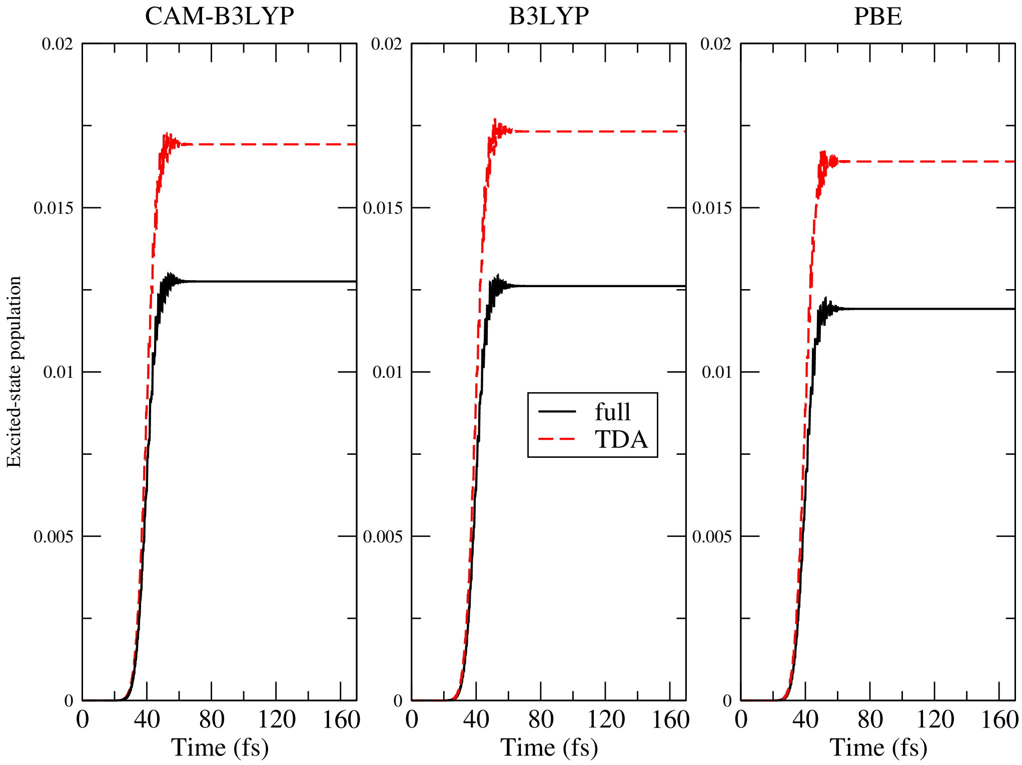

FIG. S2. Comparison between TDDFT and TDDFT/TDA time-evolution of the population of the first bright excited state of HBDI for CAM-B3LYP, B3LYP, and PBE exchange-correlation functionals. Results are reported for a delay time Δt = 10 fs between the two pulses and FWHM=15 fs.

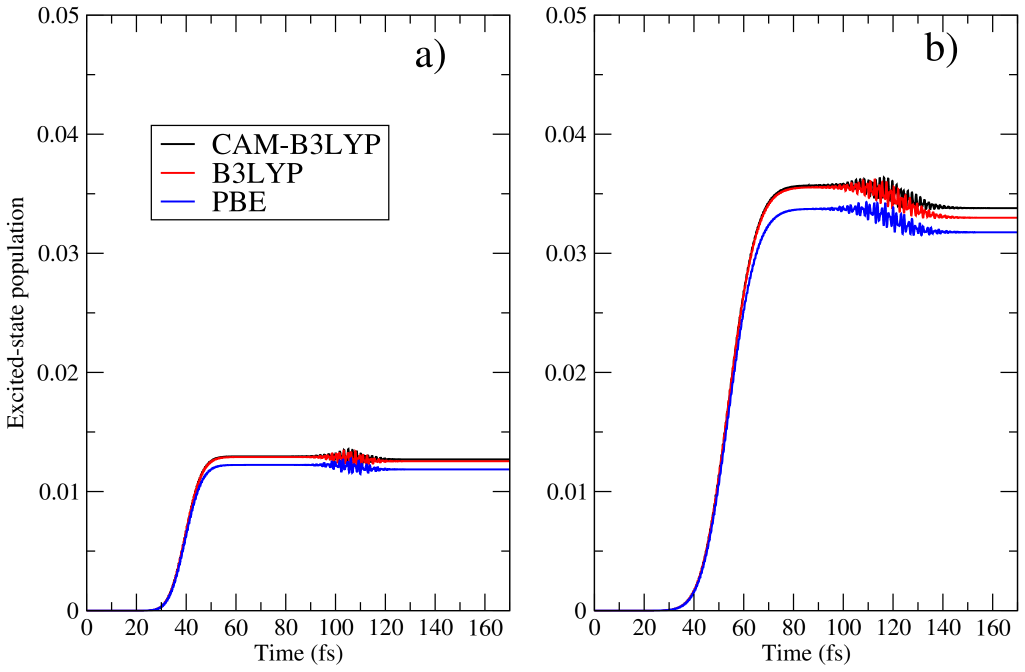

FIG. S3. Full TDDFT time-evolution of the population of the first bright excited state of HBDI for CAM-B3LYP, B3LYP, and PBE exchange-correlation functionals. Results are reported for a delay time Δt = 70 fs between the two pulses and a) FWHM=15 fs, and b) FWHM=25 fs.

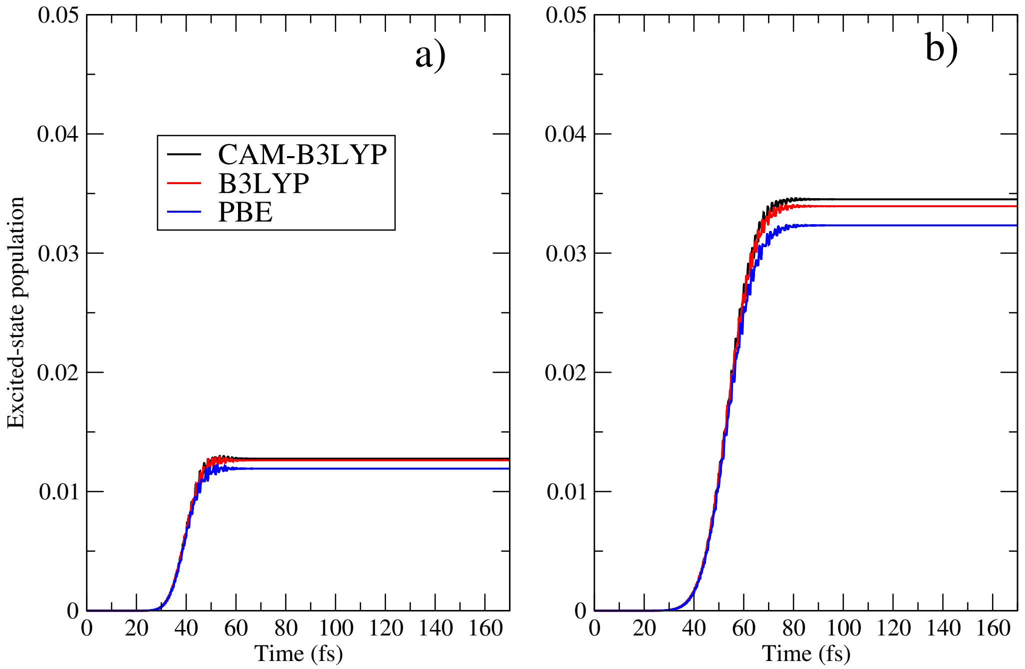

FIG. S4. Full TDDFT time-evolution of the population of the first bright excited state of HBDI for CAM-B3LYP, B3LYP, and PBE exchange-correlation functionals. Results are reported for a delay time Δt = 10 fs and a) FWHM=15 fs b) and FWHM=25 fs.

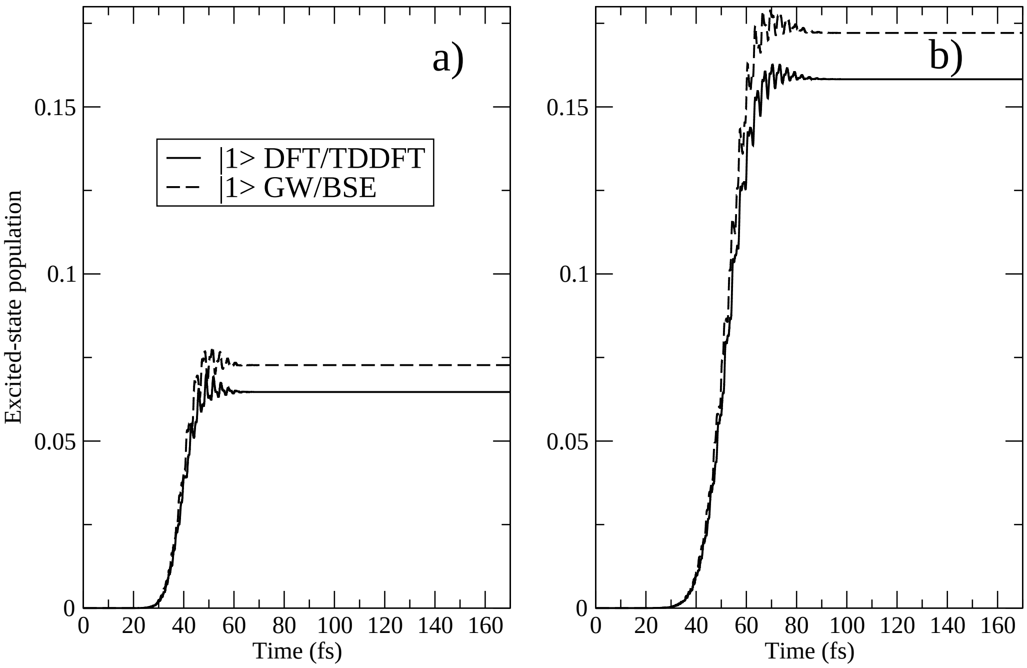

FIG. S5. Comparison between TDDFT/TDA/CAM-B3LYP and BSE time-evolution of the population of the first bright excited state of DNQDI. Results are reported for a delay time Δt = 10 fs and a) FWHM=15 fs and b) FWHM=25 fs.

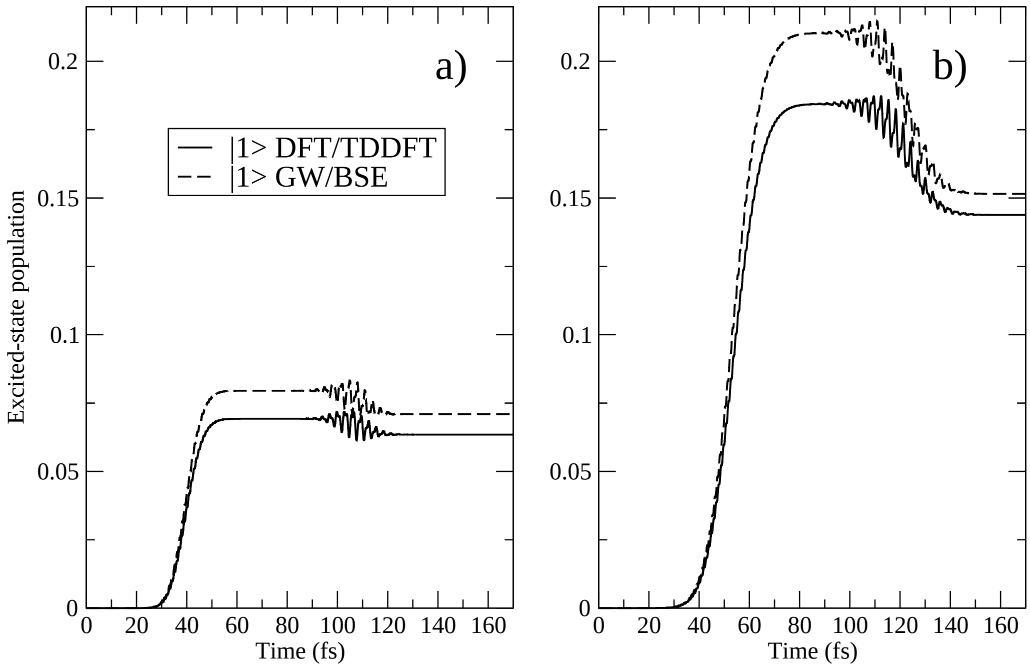

FIG. S6. Comparison between TDDFT/TDA/CAM-B3LYP and BSE time-evolution of the population of the first bright excited state of DNQDI. Results are reported for a delay time $\Delta t = 70$ fs and a) FWHM=15 fs and b) FWHM=25 fs.

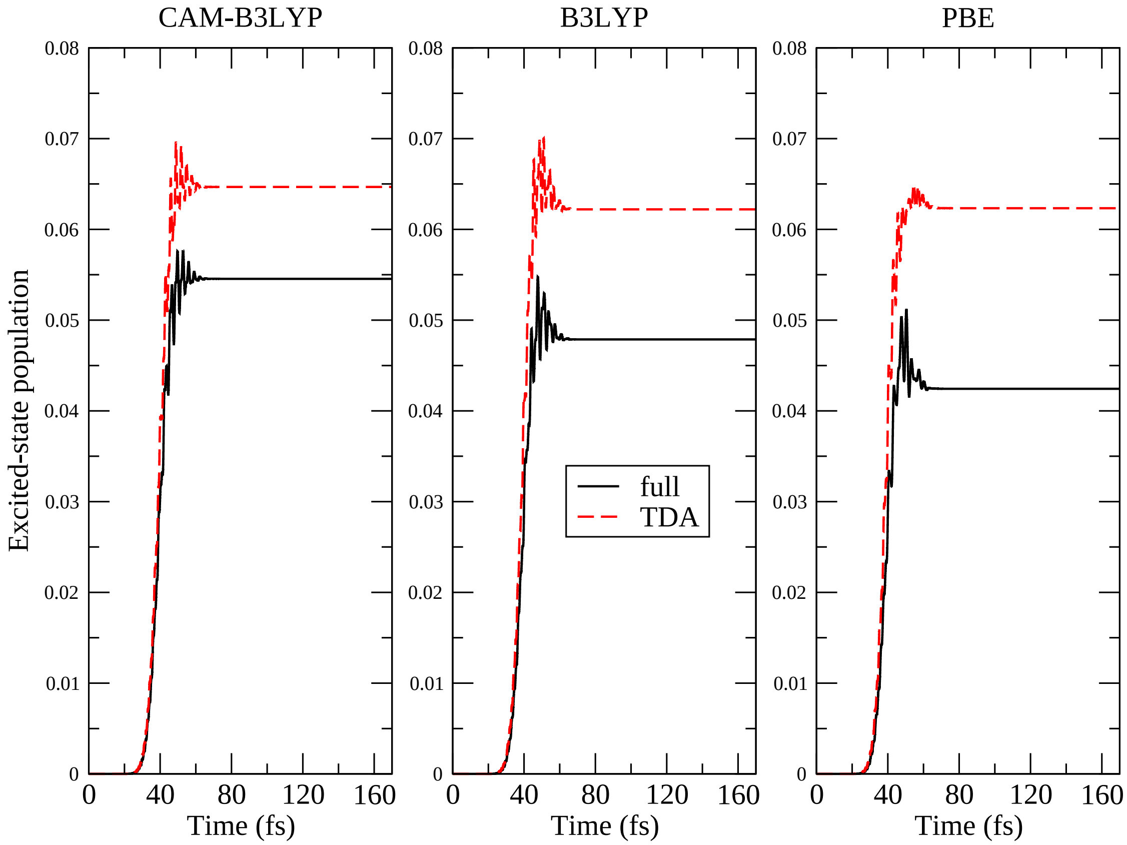

FIG. S7. Comparison between full TDDFT and TDDFT/TDA time-evolution of the population of the first bright excited state of DNQDI for CAM-B3LYP, B3LYP, and PBE exchange-correlation functionals. Results are reported for a delay time Δt = 10 fs and FWHM=15 fs.

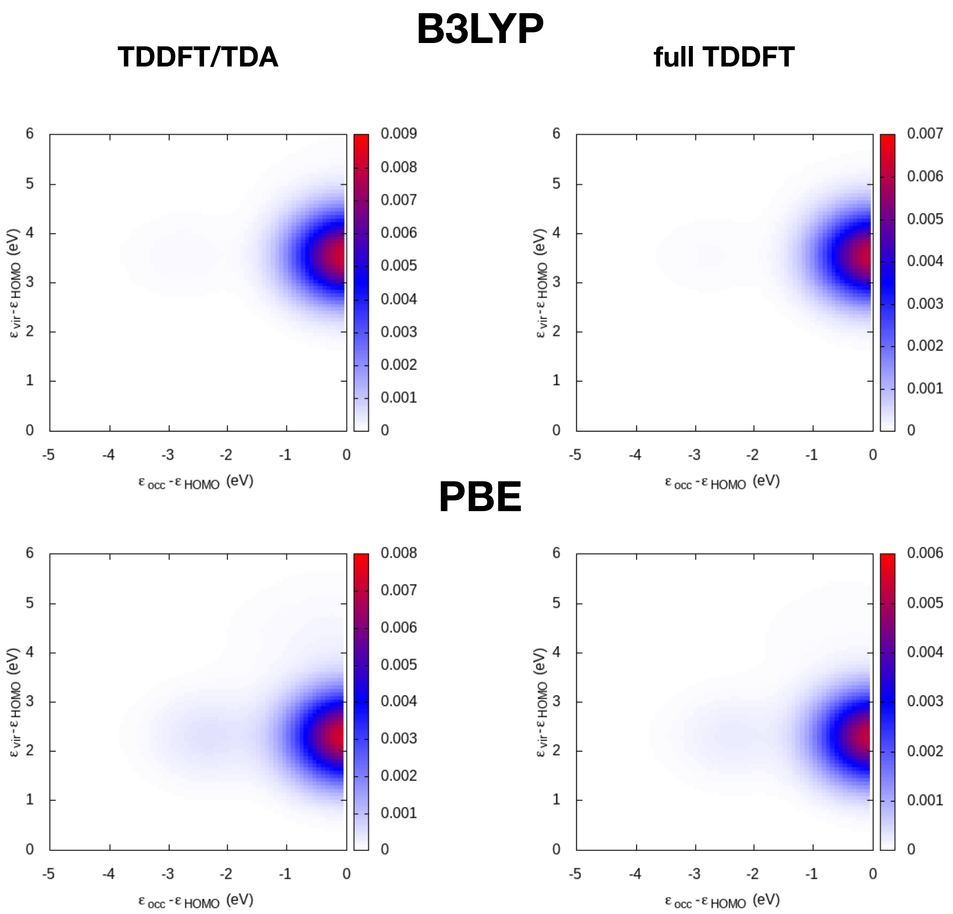

FIG. S8. Time-resolved TCM of HBDI at 36 fs using TDDFT/B3LYP and TDDFT/TDA/B3LYP (upper panels), and TDDFT/PBE and TDDFT/TDA/PBE (lower panels).

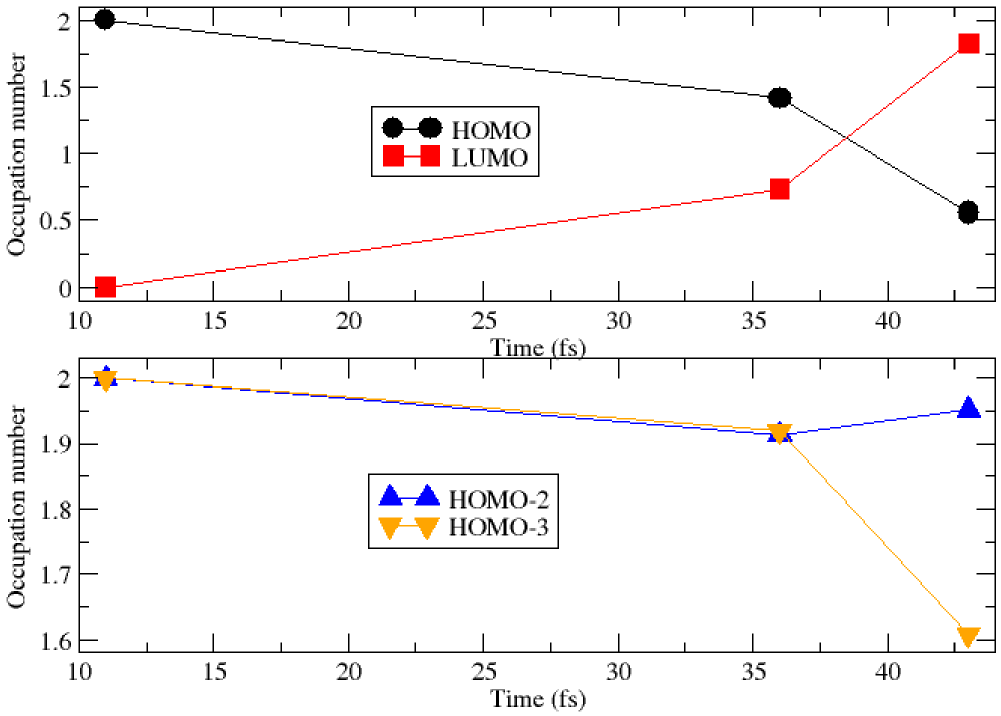

FIG. S9. Time evolution of MOs occupation number related to the HBDI electron dynamics with $I = 5 \times 10^{12}$ W/cm^2. Snapshots discussed in the main text are shown.

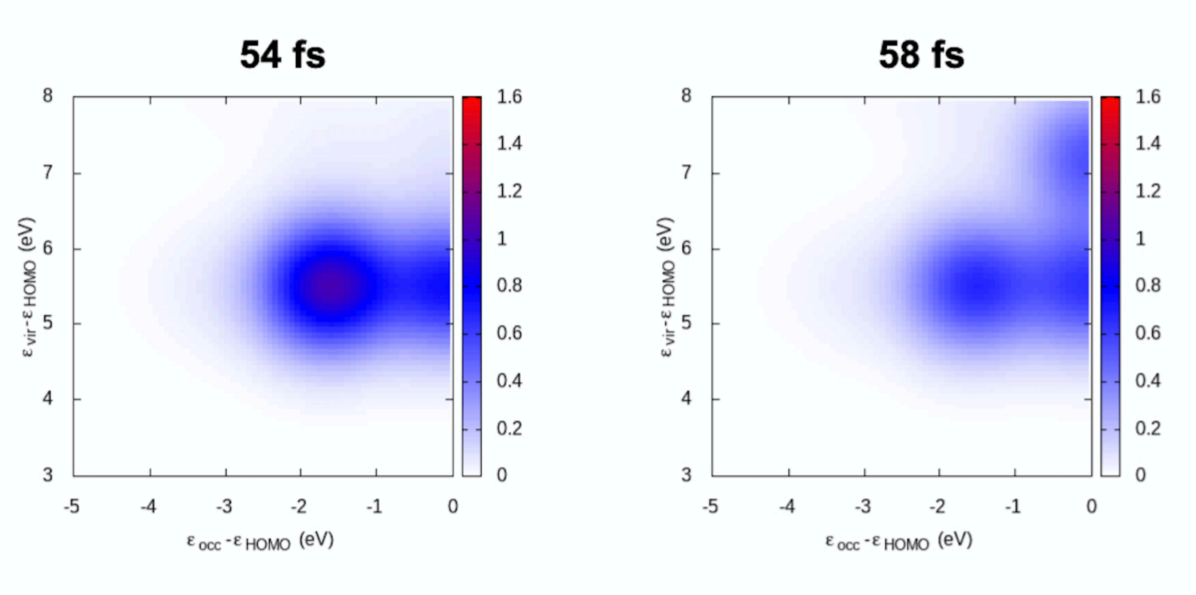

FIG. S10. Time-resolved TCM of HBDI at TDDFT/CAM-B3LYP level, at 54 and 58 fs using intense pulses (see main text for details).

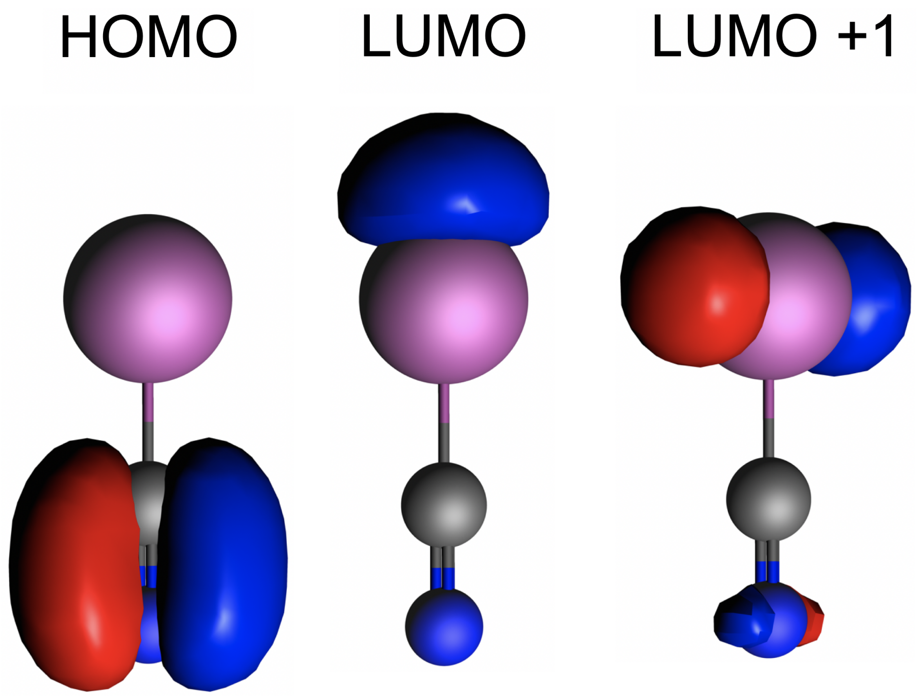

FIG. S11. Plots of HOMO, LUMO, and LUMO+1 of the LiCN molecule calculated at TDDFT/CAM-B3LYP level of theory.

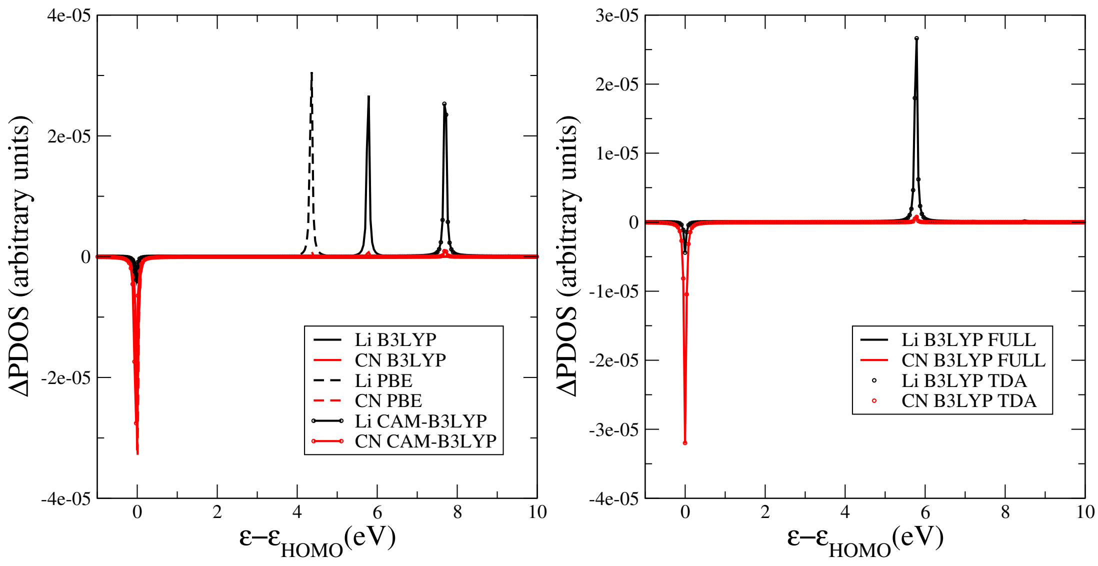

FIG. S12. ΔPDOS for Li and CN fragments of LiCN at 48.4 fs. Left: full TDDFT results for different exchange-correlation functionals. Right: comparison between full TDDFT and TDA results by using the B3LYP exchange-correlation functional.

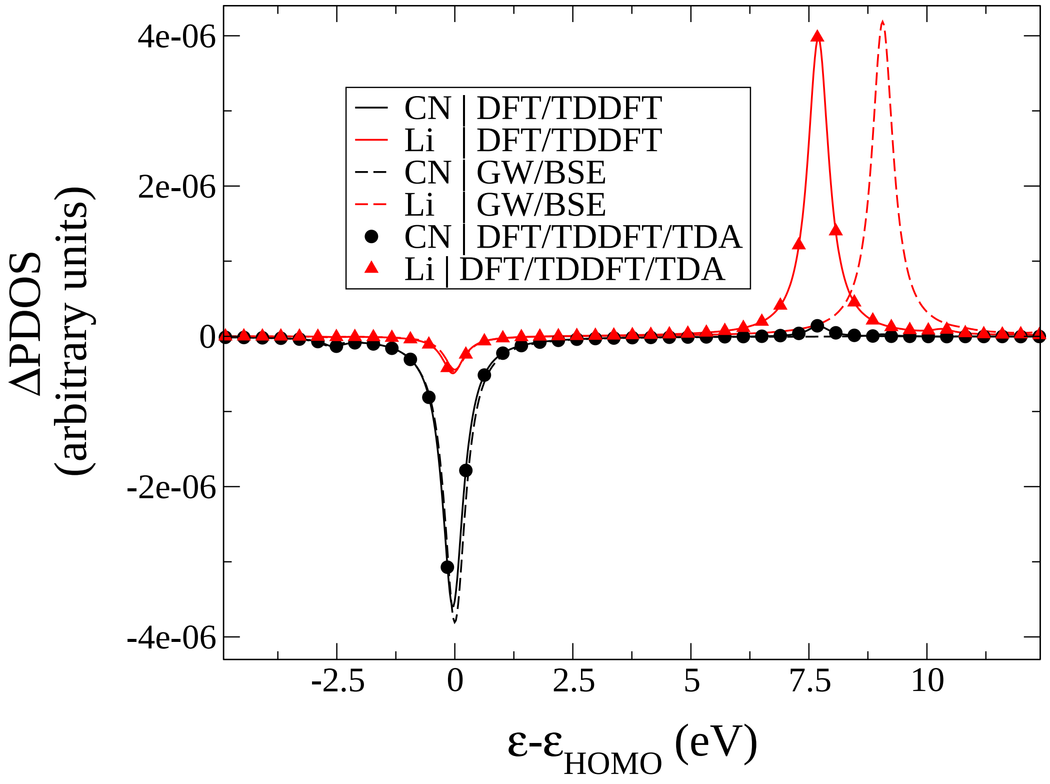

FIG. S13. Comparison of ΔPDOS for Li and CN fragments of LiCN at 48.4 fs for GW/BSE (dashed),DFT/TDDFT (solid), DFT/TDDFT/TDA (symbols) levels of theory. Full TDDFT and TDDFT/TDA results are obtained with the CAM-B3LYP functional.

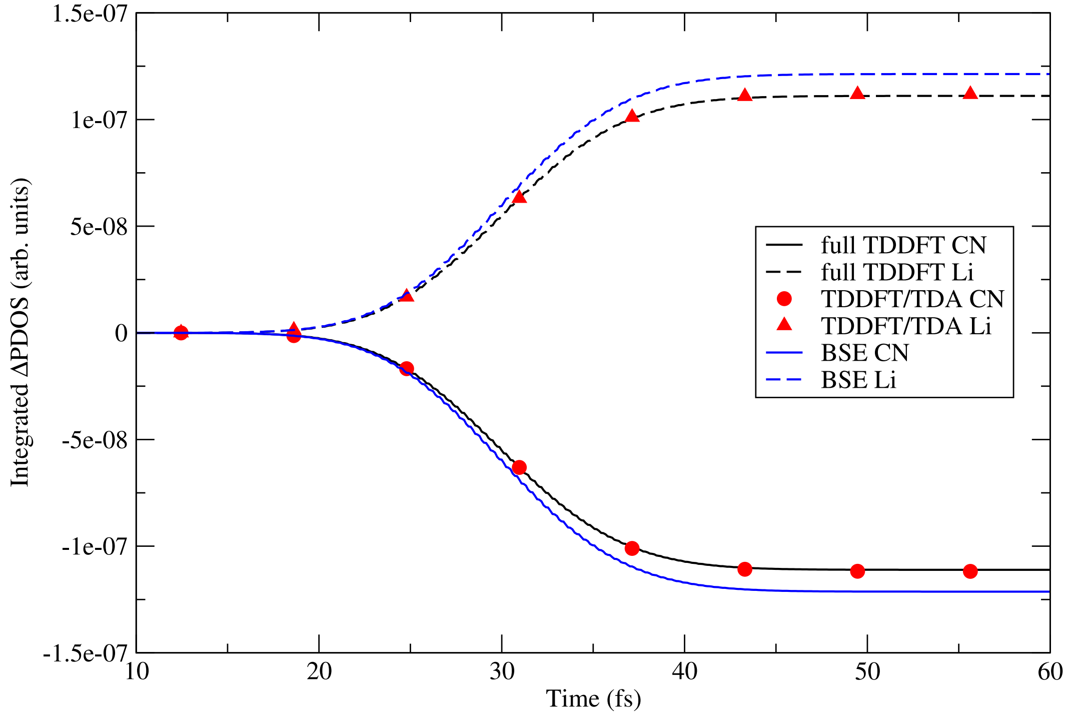

FIG. S14. Energy-integrated ΔPDOS of LiCN for the $|0\rangle \rightarrow |1\rangle$ transition at full TDDFT/CAM-B3LYP, TDDFT/TDA/CAM-B3LYP and BSE level of theory.

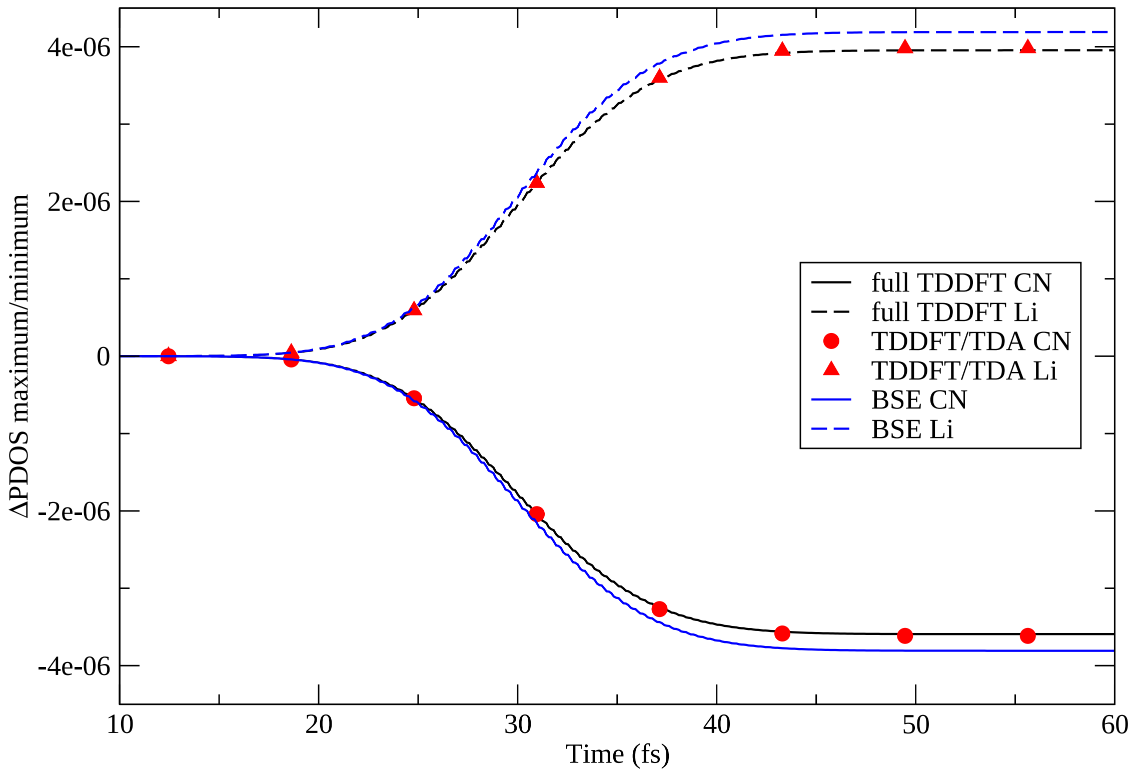

FIG. S15. Time evolution of maximum/minimum of LiCN ΔPDOS for the $|0\rangle \rightarrow |1\rangle$ transition at full TDDFT/CAM-B3LYP, TDDFT/TDA/CAM-B3LYP and BSE level of theory.
